# Photodimerization of Ferroelectric *N*,*N*′-Ditetradecyl-stilbenediamide Derivative

**DOI:** 10.1021/jacs.5c00346

**Published:** 2025-02-24

**Authors:** Yunya Zhang, Takashi Takeda, Tomoyuki Akutagawa

**Affiliations:** † Graduate School of Engineering, Tohoku University, 6-6-07 Aramaki Aza Aoba, Aoba-ku, Sendai 980-8579, Japan; ‡ Institute of Multidisciplinary Research for Advanced Materials (IMRAM), Tohoku University, 2-1-1 Katahira, Aoba-ku, Sendai 980-8577, Japan; § Department of Chemistry, Faculty of Science, Shinshu University, 3-1-1 Asahi, Matsumoto 390-8621, Japan

## Abstract

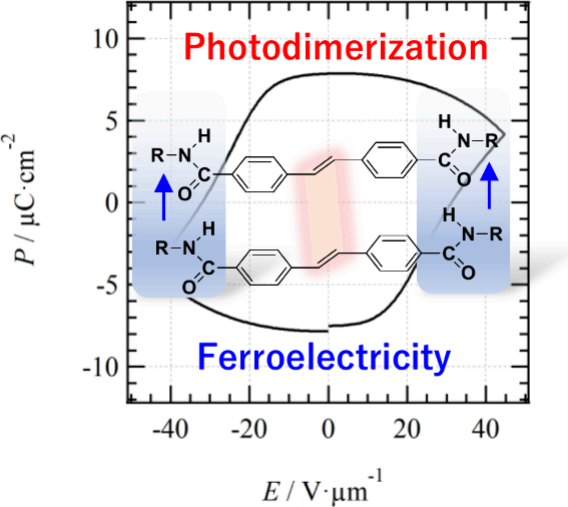

A solid state [2 + 2] photodimerization reaction of —C=C— bonds in solids has been designed by controlling the molecular arrangement using supramolecular chemistry. Stilbene derivative (**C14SDA**) with alkyl amide chains (—CONHC_14_H_29_) forms intermolecular amide hydrogen-bonding chains and exhibits reversible successive phase transitions (S1 → S2 → S3 → L) corresponding to the dynamics of the alkyl chains. In the high-temperature solid phase S3, the alkyl chains partially melt, resulting in a one-dimensional (1D) dynamic intermolecular amide hydrogen bond and ferroelectric behavior with hysteresis in the electric field–polarization curve due to polarization reversal of the dipole moment by an external electric field. The S1 phase of **C14SDA** did not exhibit a photodimerization reaction, while the dynamic S2 and S3 phases exhibited a [2 + 2] photodimerization reaction to form reaction products with cyclobutane rings. In the dynamic ferroelectric S3 phase, *trans*–*cis* isomerization of stilbene was observed simultaneously with the formation of the photodimerization product. When the photodimerization reaction was attempted with an electric field applied to the S3 phase, thermal molecular fluctuations were suppressed by the electric field, increasing the distance between —C=C— double bonds and reducing the photoreaction yield.

## Introduction

There are light-responsive functional π-molecules that change their molecular structure and electronic state in response to light.^1−3^ Azobenzene and stilbene derivatives are known to show *trans*- to *cis*-photoisomerization at the —N=N— and —C=C— double bonds at the molecular center upon photoirradiation.^4−12^ The *trans*–*cis* isomerization reactions of azobenzenes and stilbenes are limited in the solid crystal because they involve large-amplitude conformational changes in the molecular structure. However, in some azobenzene derivatives, an exception is observed in the *trans*–*cis* photoisomerization reaction in the crystalline state caused by light irradiation, and interesting mechanical responses of the single-crystal have been reported.^13−18^ In the crystal structures, the degree of freedom of molecular motion is limited, and there are restrictions on the structural changes and physical property responses by photoirradiation. On the other hand, chemical reactions in solids induced by light irradiation have long been known. For example, when the —C=C— double bonds of cinnamates are arranged at the nearest neighboring position to each other in a crystal, a [2 + 2] cycloaddition reaction by photodimerization is known to occur upon light irradiation.^19−21^ The condition for such a photodimerization reaction is that the distance (*d*_C=C_) between the —C=C— double bonds must be less than 4.2 Å, which is known as Schmidt’s rule.^22^ In the solution phase, [2 + 2] photodimerization reactions can form four kinds of regio- and stereospecific products of *syn*-HH, *anti*-HH, *syn*-HT, and *anti*-HT, whereas, in the solid state, the structure of the photodimerization product depends on its molecular arrangement in a crystal.^23−25^ Therefore, the control of photoreactions in solids depends on how to control the molecular arrangement in the crystals. Recently, nanospaces formed by metal–organic frameworks (MOFs) have attracted much attention as a new crystalline space for photodimerization reactions in solids. MOFs can provide a reaction environment different from that of ordinary crystalline spaces, offering new possibilities for controlling solid-state reactions.^26−37^ For example, Matsuda et al. reported that photodimerization reactions occur in the nano space formed by MOFs even when the distance between adjacent —C=C— bonds is *d*_C=C_ = 5.9 Å, a significant deviation from Schmidt’s rule.^38^ Photodimerization reactions and polymer synthesis in MOF nano spaces have also been reported, and studies focusing on more dynamic crystalline photoreaction environments, as opposed to photoreactions in general static crystal lattices, have been attracting much attention.^39−42^ More recently, structurally controlled photodimerization reactions of Werner-type metal-coordination complexes have been reported, and solid-phase photoreactions using new crystalline fields have attracted much attention.^43^

Dynamic molecular assemblies are interesting research targets from the viewpoint of controlling physical properties.^44,45^ Organic ferroelectrics have been developed focusing on the dynamics of proton transfer, ionic displacement, molecular rotation, and molecular displacement in various molecular assemblies such as liquid crystals and crystalline materials.^46−50^ We have focused on alkylamide chains (—CONHC_*n*_H_2*n*+1_) and have attempted to develop novel organic ferroelectrics by introducing them into various functional π-electronic frameworks.^51−66^ When multiple alkylamide chains are introduced into a simple benzene derivative, intermolecular amide N—H···O= hydrogen bonds form one-dimensional (1D) polar chains, and the application of an external electric field induces a collective inversion of the polar amide hydrogen bonds, resulting in hysteresis in the electric field–polarization (*P*–*E*) curve. In crystals containing long alkyl amide chains, where there is a successive solid–solid phase transition with increasing temperature, the partial melting of alkyl chains in the high-temperature solid phase causes inversion of the amide hydrogen bond, resulting in ferroelectricity.^54,55^ In porphyrin,^65^ pyrene,^52,66^ and azobenzene derivatives with alkylamine chains,^56^ multifunctional materials can be obtained in addition to ferroelectricity, luminescence, and characteristic optical absorption properties. In alkylamide-substituted azobenzene derivatives, *trans*–*cis* photoisomerization reactions were expected to be realized; however, in the molecular assembly structures in liquid crystals and solids, no significant changes in molecular structure upon photoirradiation were realized.^56^

**Scheme 1:**
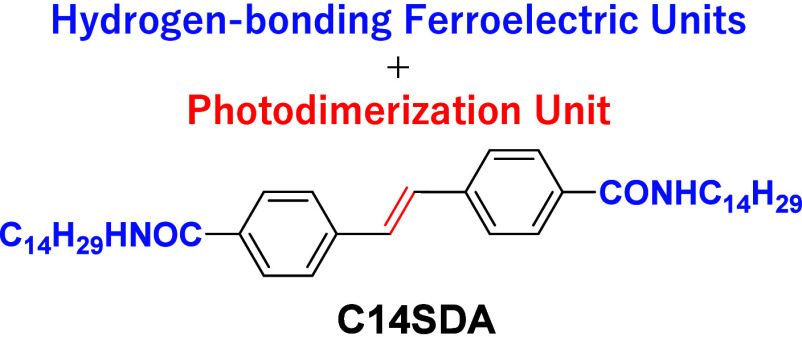
Chemical Structure of **C14SDA** Intermolecular N—H···O= hydrogen-bonding units and a photodimerization unit coexisted in a molecule.

We focused on the [2 + 2] photodimerization reaction of the —C=C— double bond of the stilbene π-core in the solid phase and reported (C_*n*_H_2*n*+1_NH_3_^+^)_2_(**SDC**^**2–**^) single crystals, in which alkylammonium with different chain lengths was combined as counter cations of stilbenedicarboxylate (**SDC**^**2–**^).^67^ A series of (C_*n*_H_2*n*+1_NH_3_^+^)_2_(**SDC**^**2–**^) with varying alkyl chain length *n* from 1 to 10 were prepared in the single crystals, and their photodimerization reaction was controlled by adjusting their molecular arrangement.^64^ Successes have also been reported in controlling the arrangement of stilbene units using intermolecular hydrogen-bonding interactions and [2 + 2] photoreactions.^68,69^ In the present study, to achieve the coexistence of ferroelectricity by introducing alkylamide chains and [2 + 2] photodimerization addition reaction, a stilbene derivative 4,4′-(ethene-1,2-diyl)bis(*N*-tetradecyl benzamide) (**C14SDA**), in which two tetradecylamide chains (—CONHC_14_H_29_) are introduced at the terminal of the molecule, was synthesized. The molecular structure of the stilbene derivative is similar to that of the azobenzene derivative, with two decylamide chains introduced at the ends of the molecule reported previously. In the azobenzene derivative, the formation of a lamellar liquid crystal phase was observed as the temperature increased, and the *P*–*E* hysteresis was observed in the liquid crystal state. In this study, we focused on **C14SDA**, in which the central structure of the molecule was changed from —N=N— to —CH=CH—, and investigated its phase transition behavior, molecular assembly structure, dielectric response, ferroelectric properties, and photodimerization reaction (Scheme 1).

## Results and Discussion

### Phase Transition Behavior

**Figure 1:**
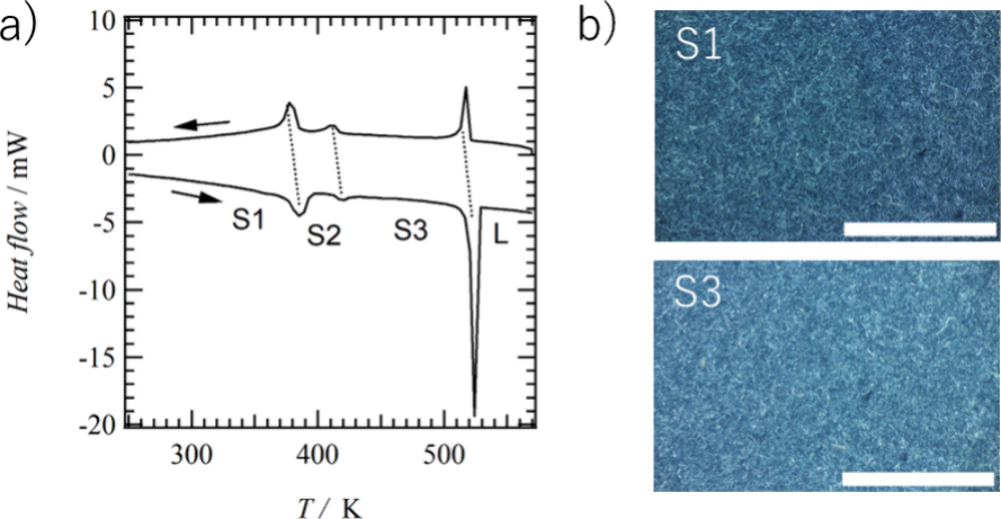
Phase transition behavior of **C14SDA**. (a) DSC chart at the temperature range from 250 to 570 K. Arrows indicate the direction of temperature change. (b) Temperature-dependent POM images at S1, S2, and S3 phases under the cross-Nicole optical arrangement. Scale bars are 500 μm.

**C14SDA** with two hydrogen-bonding sites showed high thermal stability in thermogravimetric (TG) measurements, showing no weight loss up to 650 K (Figure S2). Azobenzene derivatives with two —CONHC_10_H_21_ chains,^56^ which have similar molecular structure to **C14SDA**, show weight loss at 570 K, indicating the high thermal stability of **C14SDA**. Figure 1a shows differential scanning calorimetry (DSC) charts in the temperature range from 250 to 570 K. The solid S1 phase around room temperature changes to the solid state S2 phase at 386 K with increasing temperature and then to the solid-state S3 phase at 419 K. Finally, the S3 phase changes to isotropic liquid (L) at 523 K. This multistep successive phase transition behavior was observed reversibly during the cooling process from the L sate. Figure 1b shows polarized optical microscope (POM) images of S1, S2, and S3 phases under a cross-Nicole optical arrangement (Figure S3). In all solid phases, birefringence was observed in the POM images, and no fluidity characteristic of liquid crystalline or plastic crystalline phases was observed. The transition enthalpy changes (Δ*H*) of the S1–S2, S2–S3, and S3–L phase transitions were 22.6, 3.2, and 64.7 kJ mol^–1^, respectively, while the transition entropy changes (Δ*S*) for the S1–S2, S2–S3, and S3–L phase transitions were 66.2, 7.6, and 123.7 J mol^–1^ K^–1^, suggesting that partial melting of the alkyl chains occurs at the S1–S2 phase transition. The intermolecular N—H···O= hydrogen bond changes to the L state after the S3–L phase transition and loses long-range order. Since a multistep solid–solid phase transition is observed, there is a thermal dynamic due to the partial melting of the crystal lattice as the temperature increases.^51,52^

### Crystal Structure of **C4SDA**

**Figure 2:**
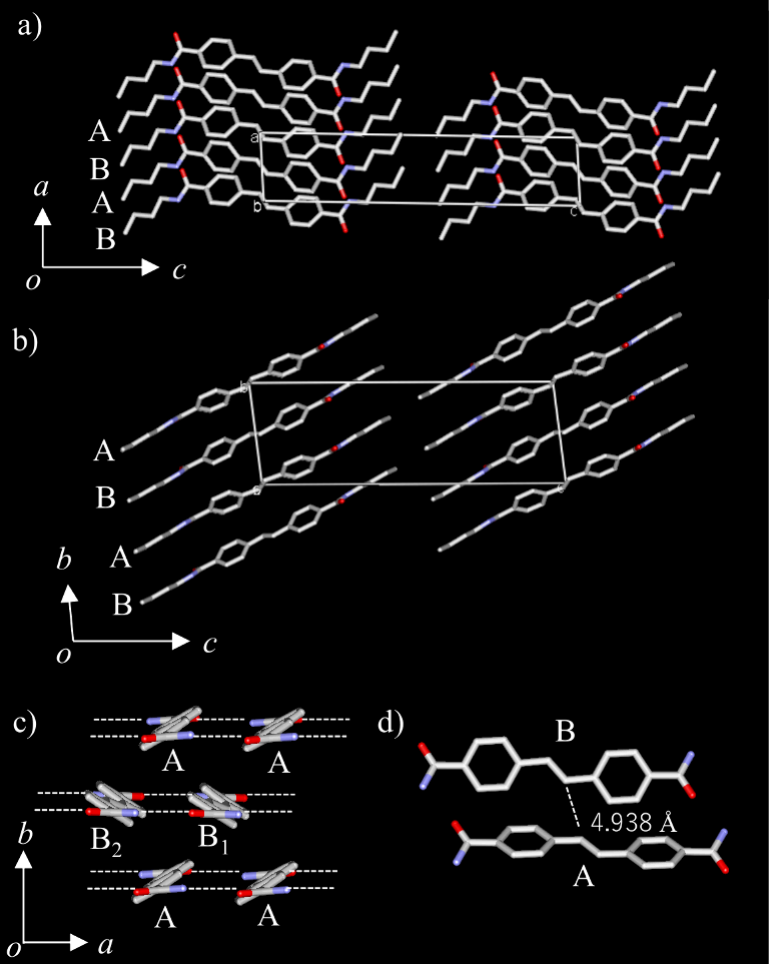
Crystal structure of **C4SDA** at 173 K. Unit cells viewed (a) along the *b*-axis and (b) along the *a*-axis. Two crystallographically independent **C4SDA** molecules (**A** and **B**) existed. (c) Double N—H···O= hydrogen-bonding chains of ∼**A**∼**A**∼ and ∼**B**∼**B**∼ along the *a*-axis. (d) Molecular arrangement between **A** and **B** with *d*_layer_ = 4.938 Å.

We also synthesized 4,4′-(ethene-1,2-diyl)bis(*N*-butylbenzamide) (**C4SDA**) with short alkyl chain length for single crystal X-ray structural analysis^70,71^ and attempted to evaluate its packing structure and intermolecular amide-type hydrogen bonds. The space group of the **C4SDA** crystal was centrosymmetrical *P*-1, with two crystallographically independent molecules (**A** and **B** in Figure S4), each with an inversion center. Figures 2a and 2b show the unit cells at 173 K viewed along the *b*- and *a*-axis, respectively. Along the *c*-axis, alkyl chains of —C_4_H_9_ and stilbene π-cores were alternately arranged to form a lamellar structure. Molecules **A** and **B** were alternately aligned along the *a*-axis, and the intermolecular hydrogen-bonding chain (N—H···O=)_∞_ formed a 1D hydrogen-bonding double chain with two amide groups at the molecular terminals (Figure 2c).

The intermolecular hydrogen bonds are arranged at the ∼**A**∼**A**∼ and ∼**B**∼**B**∼ array, with a N—H···O= hydrogen-bond distance (*d*_N—O_) of 3.0233(2) and 3.0013(3) Å, respectively. The stilbene moieties of **C4SDA**s were arranged in the order ∼**A**∼**B**∼**A**∼**B**∼ along the *b*-axis, and the overlap mode of molecules **A** and **B** was different from the typical π–π manner with aligned stilbene planes, in which their π-planes were twisted about 116° with each other (Figure 2d). The center-to-center distance (*d*_C=C_) of the nearest neighboring —C=C— bonds was 4.938 Å, which was 0.7 Å longer than the possible formation distance *d*_layer_ = 4.2 Å for the photodimerization reaction by Schmidt’s rule. Therefore, there are no significant intermolecular interactions between **A** and **B** molecules at 173 K in **C4SDA** that would permit photodimerization reactions, suggesting that photodimerization reactions may not occur only between **A**∼**B** pairs, whose π-planes are twisted toward each other.

### The Molecular Assembly Structure of **C14SDA**

**Figure 3:**
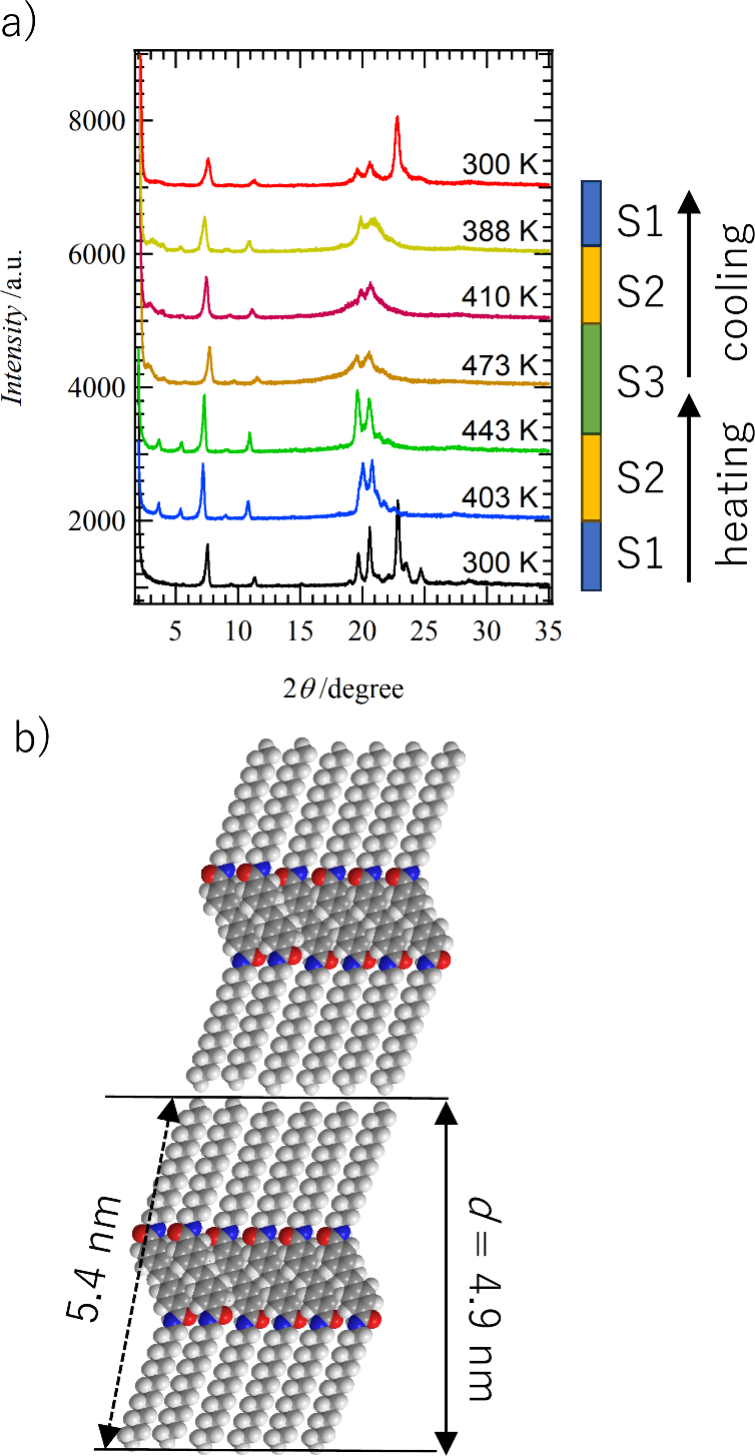
Temperature-dependent change of molecular assembly structures of **C14SDA**. (a) PXRD pattern at the heating and cooling processes for S1, S2, and S3 phases. (b) Schematic layered molecular assembly structure of **C14SDA** with a *d*_layer_ spacing of 4.9 nm (PXRD) and an estimated molecular length of **C14SDA** assuming all-staggered alkyl chains (5.4 nm).

Since it was difficult to obtain a high-quality single crystal of **C14SDA** with long alkyl chains, powder X-ray diffraction (PXRD) was used to discuss the molecular assembly structure based on the crystal structure of **C4SDA** at 173 K. **C14SDA** shows a reversible phase transition from the S1 phase to the S2 and S3 phases by increasing temperature. Figure 3a shows the temperature-variable PXRD patterns of **C14SDA**. The diffraction peaks of the S1 phase at 300 K were sharp and highly crystalline. The sharp diffraction peak observed at 2θ = 7.55° corresponds to the length of the *c*-axis found in the **C4SDA**, i.e., the periodicity of the layered structure, and its correlation distance is *d*_layer_ = 4.68 nm (see Table S2). Assuming that the two —C_14_H_29_ chains of **C14SDA** are in an all-staggered conformation, the maximum molecular length of **C14SDA** is 5.4 nm. It is considered to be oriented with the molecular long axis of **C14SDA** tilted at about 60° to the layer *ab*-plane (Figure 3b). Similar to the molecular arrangement of **C4SDA**, the layered structure formed by the alternation of the two alkyl chains and the stilbene π-cores is in good agreement with the *d*_layer_ spacing of **C14SDA**. After the phase transition from S1 to the S2 phases with increasing temperature, the diffraction peaks originating from the layered periodicity become more distinct in the PXRD pattern at 443 K, and sharp Bragg reflection peaks appear at 2θ = 3.62, 5.37, 7.20, and 10.78°, whose interlayer periodicity is in good agreement with *d*_layer_ = 4.91 nm. The sharp reflection peaks at around 2θ ∼ 23° at the S1 phase disappeared, indicating a decrease in the crystalline periodicity within the layers. The tilted angle of the molecular long-axis of **C14SDA** changed from 60° in the S1 phase to 65° in the S2 phase, and at the same time, the in-plane crystallinity decreased.

Upon further temperature increase to the S3 phase at 473 K, the layered structure corresponding to *d*_layer_ = 4.9 nm is maintained, and the diffraction peak around 2θ ∼ 20° becomes broadened. This corresponds to partial conformational disorder due to the thermal motion of the long alkyl chains, suggesting a significant decrease in the crystal periodicity within the layer. Since no phase transition to the liquid crystalline phase is observed, the layered structure formed by the intermolecular N—H···O= hydrogen bonds is maintained, and the molecular arrangement is reconstructed by partial melting of the alkyl chains. **C14SDA** forms a lamellar structure with alkyl chains arranged in a head-to-head configuration and changes to the S2 and S3 phases as the temperature increases. The thermal motion of the alkyl chains causes a lowering in the periodicity within the layer and affects the intermolecular N—H···O= hydrogen bonds. As a result, the motion of polar amide groups is also associated with the temperature-dependent dielectric constants.

### Dielectric Response of **C14SDA**

**Figure 4:**
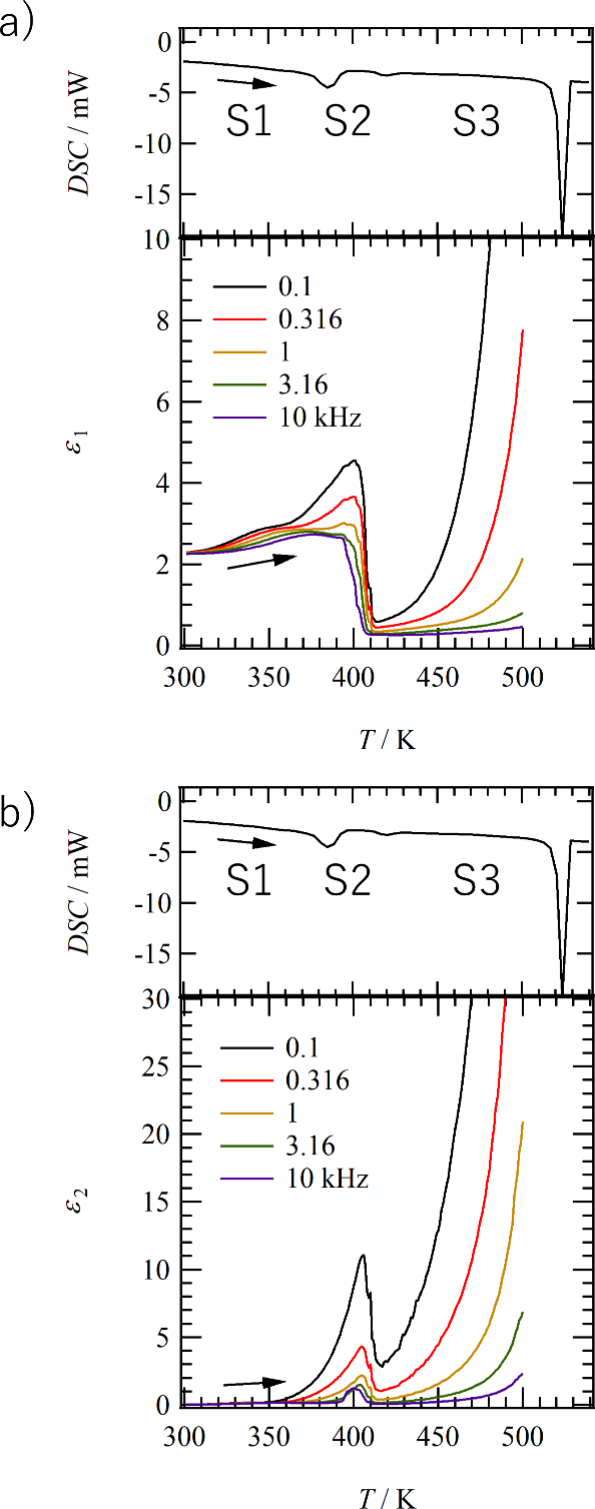
Temperature- and frequency-dependent dielectric responses of **C14SDA**. (a) Real part dielectric constant ε_1_ and (b) imaginary part dielectric constant ε_2_ with a DSC trace in the heating process.

**C14SDA** exhibits a reversible successive phase transition from the S1 to S2 to S3 to L phase, which is related to the dynamics of alkyl chains in the hydrogen-bonding layered structure. Dielectric constants are a sensitive technique to the dynamics of polar structural units in molecular assemblies and can detect the motional freedom of the polar amide hydrogen bonds in **C14SDA**. Figures 4a and 4b show the temperature- and frequency-dependent real (ε_1_) and imaginary (ε_2_) parts of the dielectric constants of **C14SDA** with compressed pellets. The DSC curves during the heating process are also shown to correspond to the changes in dielectric constant associated with the phase transition. The ε_1_ showed a characteristic change when transforming from the S1 phase to the S2 and S3 phases. In the S1 phase, the ε_1_ value was about 2, while near the transition temperature to the S3 phase, the value increased to ε_1_ = 4.5 at a low-frequency measurement of 0.1 kHz. The change in ε_1_ observed near the transition temperatures from the S2 to S3 phases is different from the frequency-dependent dielectric relaxation phenomenon, in which a frequency-independent change is observed near 405 K. Therefore, an order–disorder-type dielectric phase transition exists. The increase in the ε_1_ values in the S3 phase corresponds to the slow motional freedom of the polar hydrogen-bonding units.

The frequency-dependent ε_2_ peaks appeared at 405 K, near the phase transition from S1 to S2 and then to S3. The absolute ε_2_ value was larger at lower frequency measurements, indicating that the dynamics of dipole moments following the slow frequency are involved in the dielectric response, and this behavior is consistent with the temperature- and frequency-dependent ε_1_ behavior. The in-plane crystallinity is lowered by the phase transition from the static S1 phase to the dynamic S2 and S3 phases, accompanied by partial melting of the alkyl chains. The dielectric response is enhanced at low frequencies in the S3 phase, because the thermal motion of the dipole moment of the polar amide group is reflected in the dielectric constant. The decrease in dielectric constant during the transition from S1 to S2 phase is due to the rearrangement of the polar hydrogen-bonding units (for example, two amide hydrogen bonds aligned antiparallel to each other), accompanied by the partial melting of the alkyl chain. The temperature-dependent IR spectra also show a change in the intermolecular amide–hydrogen bond (Figure 6a).

### Ferroelectricity of **C14SDA**

**Figure 5:**
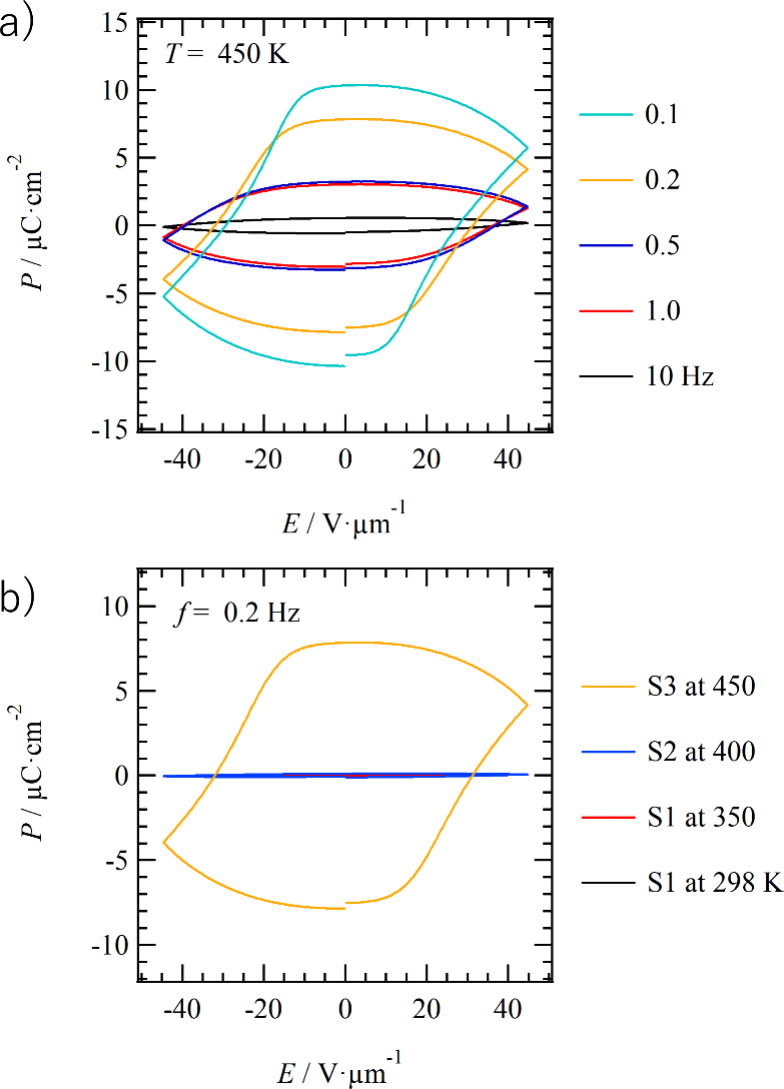
Ferroelectric response of **C14SDA**. (a) Frequency-dependent *P*–*E* curves at 450 K (S3 phase) for *f* = 0.1, 0.2, 0.5, 1.0, and 10 Hz. (b) Temperature-dependent *P*–*E* curves at *f* = 0.2 Hz for *T* = 298 and 350 K (S1 phase), 400 K (S2 phase), and 450 K (S3 phase).

The temperature- and frequency-dependent dielectric constants of **C14SDA** indicate the presence of dynamic polar amide units in both of the S3 phases. Intermolecular N—H···O= hydrogen bonds are 1D double-chain structures whose polarization inversion achieves macroscale dipole moment inversion and shows a phase transition to a ferroelectric state. The polarization inversion dynamics in ferroelectrics appear as a hysteresis in the *P*–*E* curve. Figures 5a and 5b show the frequency-dependent *P*–*E* curves at 450 K and the temperature-dependent *P*–*E* curve at 0.2 Hz, respectively. At 450 K after the phase transition to the S3 state, the dielectric constant increases significantly at low frequencies, indicating the presence of slow dipole dynamics and showing ferroelectric hysteresis behavior, with values of remanent polarization (*P*_r_) and coercive electric field (*E*_c_) at 0.2 Hz of 7.8 μC cm^–2^ and 31 V μm^–1^, respectively. As the measured frequency in the *P*–*E* curve is increased, the *P*_r_ values decrease, and the inversion motion of the intermolecular 1D double N—H···O= hydrogen bonds cannot follow the higher frequency condition. Similar ferroelectricity in the high-temperature solid-state phase has been observed in alkylamide-substituted 1,4-benzene^53,54^ and azobenzene derivatives^56^ with similar hydrogen-bonded 1D double-chain structures. In the high-temperature solid phase, where the alkyl chains are partially melted, ferroelectrics with a similar polarization inversion mechanism are possible.

### Dipole Inversion of (N—H···O=)_∞_ Chain

**Figure 6:**
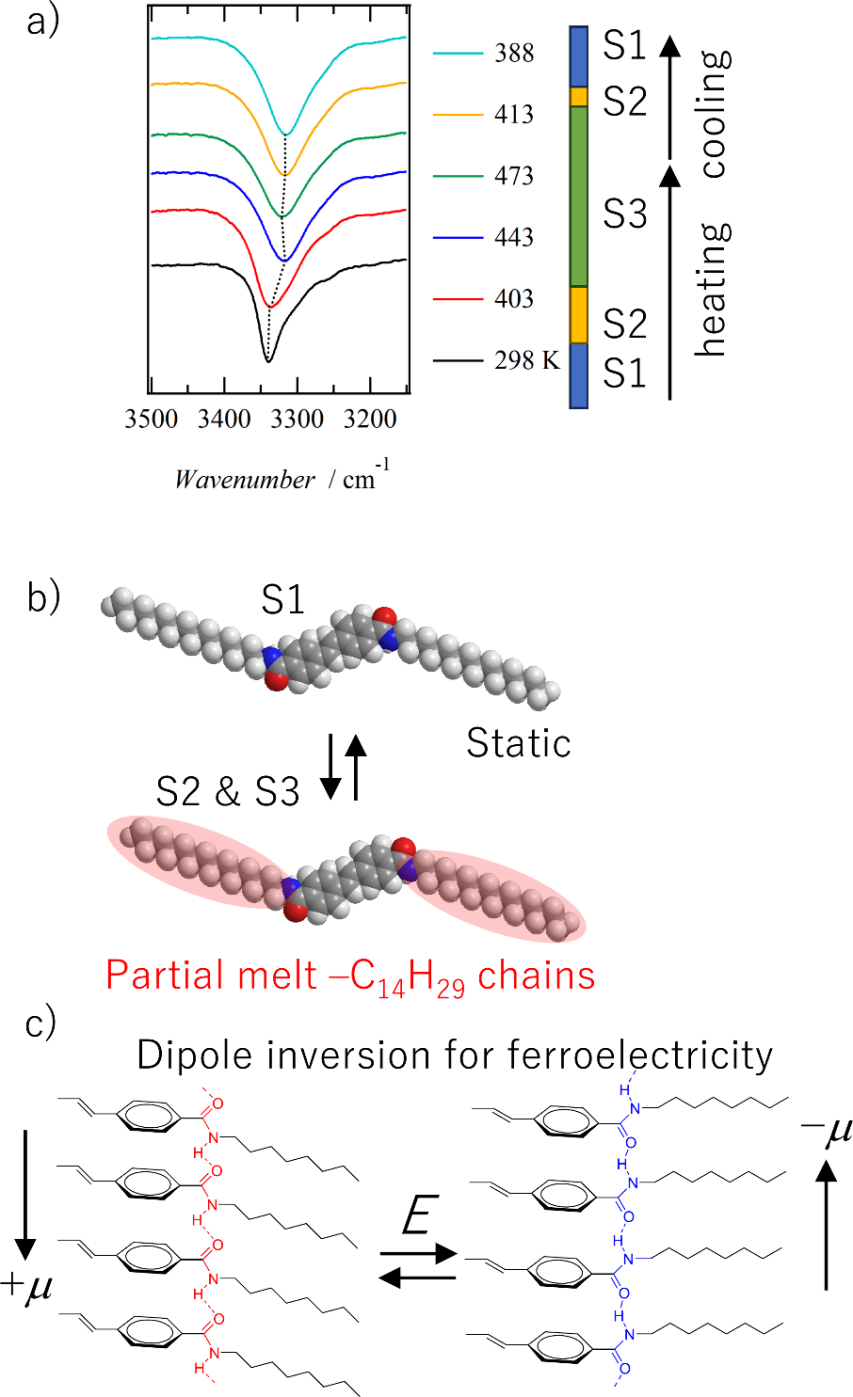
Dynamics of double (N—H···O=)_∞_ 1D hydrogen-bonding chains in **C14SDA**. (a) Temperature-dependent vibrational spectra of the ν_N—H_ band at a KBr pellet. (b) Partial melting of the —C_14_H_29_ chain conformation at S2 and S3 phases. (c) Dipole inversion of the direction of the polar (N—H···O=)_∞_ hydrogen-bonded chains by the application of the electric field (***E***). The μ is a dipole moment of a 1D chain.

The dynamics of intermolecular 1D N—H···O= double hydrogen-bonding chains were evaluated from temperature-variable IR spectra. Figure 6a shows the temperature-dependent N—H asymmetric stretching vibrational band (ν_N—H_) of **C14SDA** measured using KBr pellets. The ν_N—H_ band is observed at 3340 cm^–1^ in the S1 phase, while the ν_N—H_ band is observed at 3316 cm^–1^ in the S3 phase with a red-shift of about 25 cm^–1^. In general, the ν_N—H_ band is known to shift to higher wavenumbers as the intermolecular N—H···O= hydrogen bonds weaken by increasing the temperatures. Therefore, the phase transition from the static S1 phase to the dynamic S3 phase is accompanied by a partial melting of the alkyl chains, which weakens the intermolecular N—H···O= hydrogen bonds and leads to a polarization inversion of the polar amide–hydrogen bond that can respond to an external electric field (Figure 6b). Melting of the alkyl chains may reduce the in-plane crystalline periodicity in the layered structure, which activates the dynamics of the amide groups. Similar changes are observed for temperature changes in the carbonyl C=O bonds involved in intermolecular N—H···O= hydrogen bonds (Figure S5). As the temperature increases, the force constant of the C=O group decreases slightly as the intermolecular amide hydrogen bond weakens, causing a change in the vibrational band.

### [2 + 2] Photodimerization Reaction in Solids

**Figure 7:**
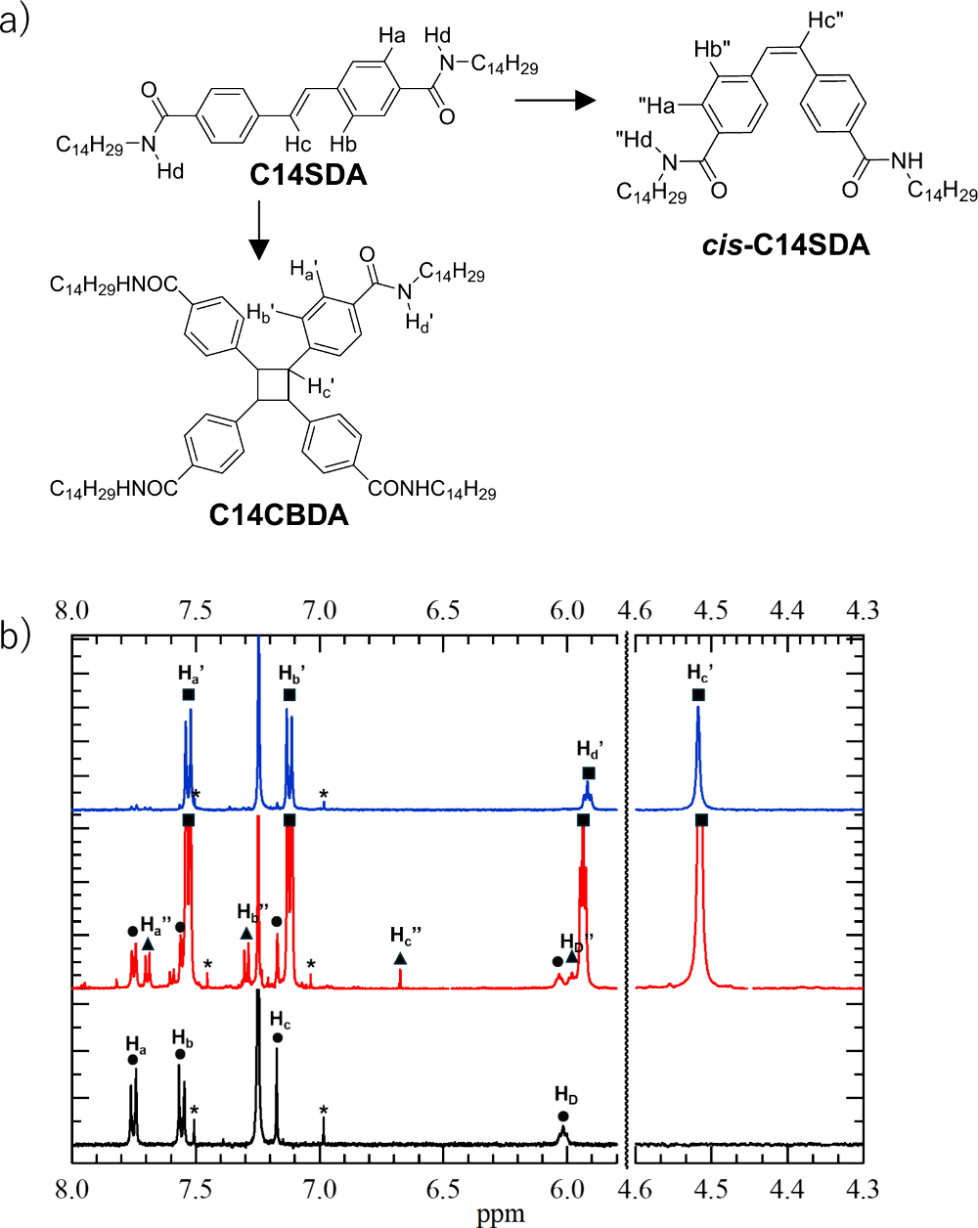
Solid state photoreaction of **C14SDA** in S1 and S3 phases. (a) Molecular structures of the photoisomerization process from *trans*-**C14SDA** to *cis*-**C14SDA** and photodimerized **C14CBDA**. (b) ^1^H NMR spectra of **C14SDA** after the 120 min photoirradiation at S1 (*T* = 298 K, black) and S3 (*T* = 470 K, red) phases and isolated **C14CBDA** (blue) in CDCl_3_. Each proton was assigned to **C14SDA** (circles), **C14CBDA** (squares), and *cis*-**C14SDA** (triangles) molecules. Asterisks (*) denote spinning sidebands.

The solid-phase photodimerization of **C14SDA**, which exhibits a successive phase transition, was investigated by irradiating **C14SDA** in solids with a wavelength of 365 nm light with 4 mW cm^–2^ (see Figure S1 in the Experimental Section). No photodimerization reaction was observed in the S1 phase of **C14SDA** at 298 K. Even in the photoirradiation of **C4SDA** at 298 K, the sample after 60 min of photoirradiation showed only the ^1^H NMR signal of **C4SDA** (Figure S6). Therefore, when the nearest neighboring —C=C— bond distance of *d*_C=C_ = 4.938 Å in single crystal X-ray structural analysis is longer than 4.2 Å, the photodimerization reaction does not occur according to Schmidt’s rule. Since the temperature range in which the S2 phase exists is narrow, the S3 phase of **C14SDA** was irradiated for *t* min (0–120 min) under controlled temperature. The reaction product was dissolved in CDCl_3_ and quantitatively analyzed by ^1^H NMR spectra. The light irradiation of stilbene derivatives in solution is known to induce *trans*–*cis* isomerization, whereas the isomerization from the *trans*- to the *cis*-form does not occur in the solid state. In the solid state, according to Schmidt’s rule, the formation of a cyclobutane ring by [2 + 2] cycloaddition reaction is governed by the arrangement of the neighboring —C=C— double bond, and when *d*_C=C_ is less than 4.2 Å, a photodimerized molecule (**C14CBDA**) is formed by dimerization reaction of **C14SDA** (Figure 7a). The chemical shifts (δ) of the H_a_, H_b_, H_c_, and H_d_ protons of *trans*-**C14SDA** are observed at *d* = 7.74, 7.56. 7.71, and 6.02 ppm (Figure S7). From the crystal structure of **C4SDA**, the *d*_C=C_ distance in the S1 phase of **C14SDA** is also estimated at *d*_C=C_ = 4.94 Å. Therefore, the [2 + 2] photodimerization reaction does not occur according to the Schmidt rule. When the S1 phase of **C14SDA** was irradiated for *t* = 120 min, no formation of dimerized **C14CBDA** was observed (black spectrum in Figure 7b). Both **C14SDA** and **C4SDA** crystals appear to have similar molecular arrangements, with intermolecular amide-type hydrogen bonds being the strongest intermolecular interactions in the molecular assemblies. The *d*_C=C_ = 4.938 Å observed in the **C4SDA** single crystal is longer than the *d*_C=C_ < 4.2 Å in Schmidt’s rule, and thus, no photodimerization reaction occurs also in **C14SDA** at 298 K. The melting of the alkyl chains of **C14SDA** at high temperatures, in addition to the onset of ferroelectricity, causes thermal fluctuations in the central stilbene units, reducing the *d*_C=C_ value. Such thermal activation of **C14SDA** results in the photodimerization reaction at the S2 and S3 phases. To confirm the importance of dynamic S2 and S3 phases, photodimerization reactions of **C4SDA** at 400, 470, and 490 K were studied under the same conditions as those for **C14SDA** (*t* = 60 min). The results showed that the photodimer formation rates at 400, 470, and 490 K were 2.7, 5.3, and 6.0%, respectively. The slight formation of photodimers may be due to the increased thermal fluctuation of the molecule with increasing temperature. The dynamic S2 and S3 phases of **C14SDA** were confirmed to be important in improving the photodimerization reaction yields (Figure S38).

On the other hand, when **C14SDA** was heated up to 470 K and irradiated for *t* = 120 min in the S3 phase, the formation of the photodimerized **C14CBDA** and *cis*-**C14SDA** was confirmed (red spectrum in Figure 7b). The formation of **C14CBDA** was confirmed by appearance of the peaks at *d* = 7.52, 7.13, 4.52, and 5.92 ppm, which were assigned to the H_a_′, H_b_′, H_c_, and H_d_′ protons of **C14CBDA**, respectively. The elemental analysis, HRMAS, and the TG measurement were consistent with the formula of **C14CBDA**·0.5(CHCl_3_) (see the Experimental Section in the Supporting Information). From the integrated values of protons, the amount of *trans*-**C14SDA** and photodimerization product **C14CBDA** formed during light irradiation time *t* can be quantified. In addition to signals for photodimerization product **C14CBDA**, signals at δ = 7.71, 7.30, 6.67, and 5.96 ppm were observed, which can be attributed to *cis*-**C14SDA**, indicating that light irradiation of **C14SDA** in the S3 phase results in the formation of both **C14CBDA** and *cis*-**C14SDA**.

**Figure 8:**
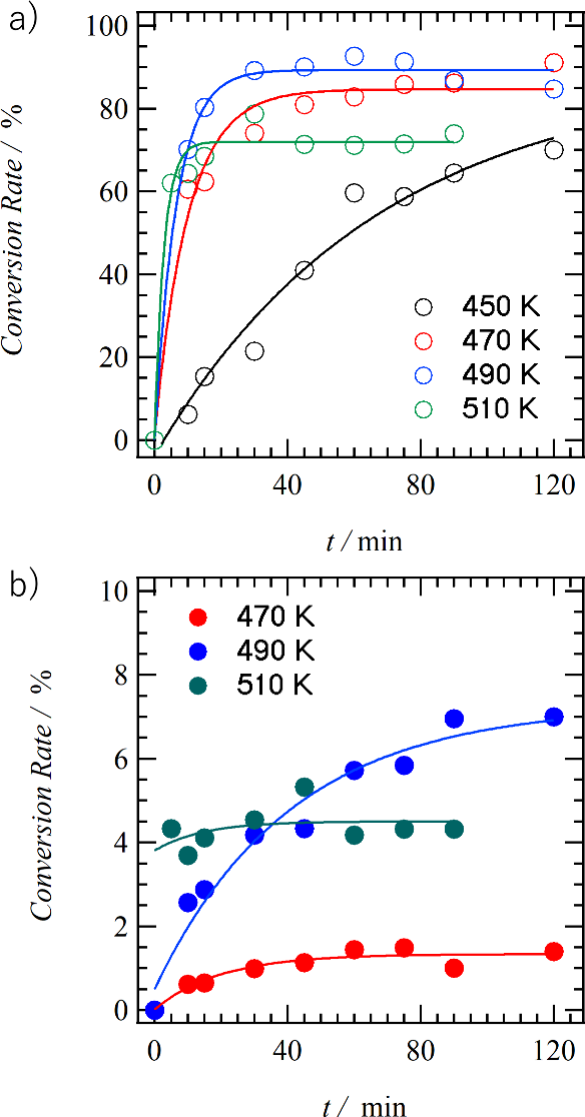
Temperature- and time (*t*)-dependent conversion ratio from **C14SDA**. (a) Formation of photodimerized **C14CBDA** at 450, 470, 490, and 510 K. (b) Formation of *cis*-**C14SDA** at 470, 490, and 510 K. All solid lines are fitted curves for reference.

Figure 8a shows the temperature and reaction time (*t*) dependence of the conversion rate from **C14SDA** to photodimerized **C14CBDA**. The formation of photodimerized **C14CBDA**, upon photoirradiation with 4 mW cm^–2^ at 450, 470, 490, and 510 K, was plotted to *t* values. The conversion ratio–*t* dependence behavior after the phase transition to the S3 phase showed temperature-dependent behavior. The conversion rate at 450 K reached about 70% after *t* = 120 min of photoirradiation (Figures S15–19), whereas the conversion rate at 470 K reached 70% after 30 min of photoirradiation (Figures S20–24). The conversion rate at 490 K reached 90% at 30 min photoirradiation (Figures S25–29). As the temperature of the S3 phase increased, photodimerization of **C14CBDA** formed after shorter periods of light irradiation time. However, when the temperature was increased to 510 K, the conversion rate reached 70% in about *t* = 20 min and did not increase further from there (Figures S30–34). In addition to the photodimerization product **C14CBDA**, the formation of *cis*-**C14SDA** in the S3 phase is also confirmed by ^1^H NMR spectra. Figure 8b shows the conversion rate of photoirradiation to *cis*-**C14SDA** for the S3 phase as a function of irradiation time *t* at 470, 490, and 510 K. The formation of *cis*-**C14SDA** is only slightly less than 10% of all products, and the formation of photodimerized **C14CBDA** is much more dominant. The conversion rate at 470 K was less than 2%, and the value changed little with increasing light irradiation time *t*. On the other hand, when the temperature was increased to 490 K, the conversion rate increased gradually depending on the irradiation time *t*, reaching about 7% at *t* = 120 min. When the temperature was further increased to 510 K, a conversion rate of about 4% was observed independent of irradiation time *t*. The temperature- and frequency-dependent dielectric constants show that the dynamics of the alkyl chains and amide hydrogen bonds are activated as the temperature increases. A temperature of 510 K is a state in which the molecular dynamics is thermally activated just before the **C14SDA** melting point, suggesting that thermal isomerization from the *trans*- to the *cis*-form occurs.

In the S3 phase, the alkyl chains melt in a lamellar structure, the molecular arrangement in the layer changes to a disordered in-plane molecular arrangement, and a ferroelectric phase is formed in which the polar amide chains can change the polarization direction by an electric field. The [2 + 2] photodimerization reaction is considered to have proceeded as a result of the dynamic change in the in-plane arrangement of molecules in the S3 phase. Such dynamics were difficult in the S1 phase, and the realization of a molecular arrangement with *d*_C=C_ < 4.2 Å according to Schmidt’s rule was achieved in the S3 phase. In the S3 phase with high kinetic degrees of freedom, dissociation of the amide hydrogen bond increased the degree of freedom of conformational change from the *trans*- to *cis*-form and may have contributed to the generation of the photoisomerization reaction. These photoreactions are consistent with the dynamics of the amide hydrogen bond as indicated by the dielectric measurements.

### Photodimerized **C14CBDA**

**Figure 9:**
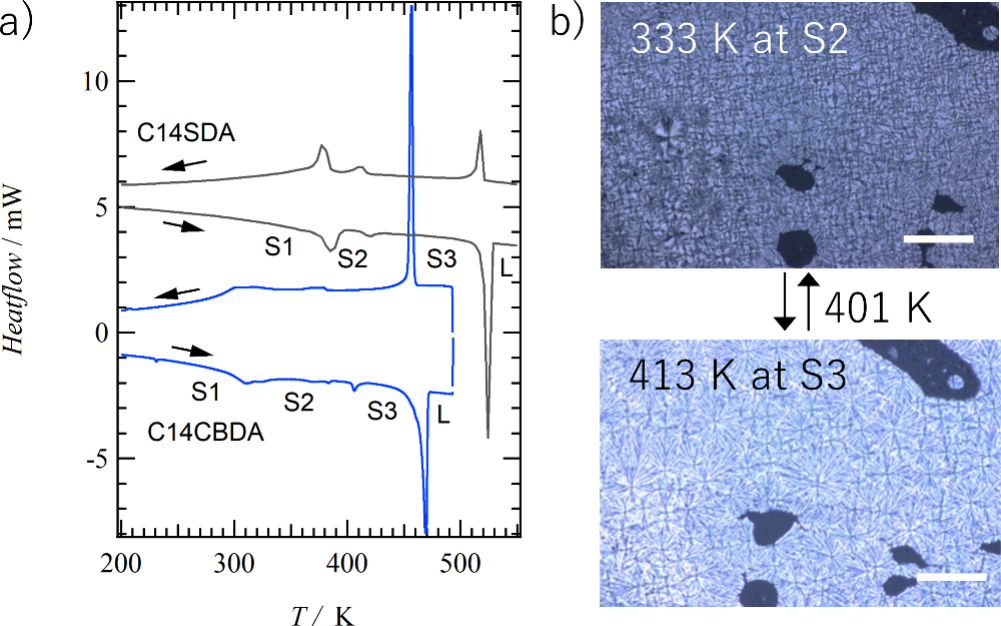
Thermal properties of **C14CBDA**. (a) DSC charts of **C14SDA** (black) and photodimerized **C14CBDA** (blue). (b) POM images of S2 and S3 phases of photodimerized **C14CBDA**. Scale bars are 500 μm.

The dimerized **C14CBDA** exhibited two-step solid–solid phase transitions at 310 and 400 K, and the melting point was observed around 455 K (Figure 9). The melting point of **C14CBDA** was 60 K lower than that of *trans*-**C14SDA** at 515 K. Therefore, the photoirradiation reaction was performed at 473 K, where **C14SDA** in the S3 phase converted to the photodimerized **C14CBDA** and to a liquid state, in which **C14SDA** partially dissolved, forming an environment with high degrees of freedom of conformational change, which may have promoted the photoisomerization to *cis*-**C14SDA**. The photodimerization reaction from *trans*-**C14SDC** to **C14CBDA** is irreversible, while the photoisomerization reaction to *cis*-**C14SDA** is generally reversible. However, due to the small amount of photoisomerization product, the details of this reaction have not yet been investigated. The decrease in the melting point can be attributed to the decrease in molecular planarity and the dissociation of intermolecular hydrogen bonds. The POM images of the S2 and S3 phases of **C14CBDA** confirmed the presence of crystallinity, with no fluidic property. In addition, a broad reflection appeared around 2θ = 20° in the PXRD pattern at 298 K in addition to the crystalline Bragg reflection (Figure S35) and the TG chart supported the existence of CHCl_3_ in the powder sample of **C14CBDA**·0.5(CHCl_3_) (Figure S36). The molecular structure of **C14CBDA** formed by photoreaction was nonplanar, with four phenyl groups extending from the central cyclobutane ring at a 90° angle to each other, which was obtained by a theoretical DFT calculation based on a B3LYP/6-31G(d) basis set (Figure S37). The IR spectra of **C14CBDA** using the KBr pellet showed a vibrational band at 3315 cm^–1^ that could be attributed to the weak amide hydrogen bonds.

When the photodimerization reaction is performed at 473 K (S3 phase), produced **C14CBDA** turns into a liquid state. The partial dissolution of **C14SDA** in the liquid state provides an environment in which conformational change is possible with a high degree of freedom, which is thought to promote the photoisomerization reaction of **C14SDA** to *cis*-**C14SDA**. It can be concluded that the coexistence of the liquid state of **C14CBDA** formed by the photodimerization reaction and the dynamic solid S3 phase of **C14SDA** increased the degree of freedom of the molecular motion of *trans*-**C14SDA** and promoted the photoisomerization reaction to *cis*-**C14SDA**.

As the high-temperature S3 phase of **C14SDA** is ferroelectric state, the photodimerization reaction under oriented macropolarization was investigated. The photodimerization reaction under an electric field was investigated at 450 K, when the alkyl chains were partially melted, and the reaction yields were compared with those in the absence of an applied electric field. The application of an electric field is thought to align the intermolecular hydrogen bonds and also to affect the orientation of the stilbene π-core. The yields of the reaction products under an electric field at 450 K were confirmed by ^1^H NMR spectra, showing that the yields decreased from around 60% in the absence of an electric field to 20–38% (Figure S38). This may be because the orientation of the thermally fluctuating —C=C— double bonds is suppressed by the electric field, resulting in an elongation of the intermolecular distance *d* between the —C=C— double bonds and a decrease in the reaction yield. In the high-temperature phase, the orientation of the π-core also thermally fluctuates with the melting of the alkyl chains, and the optimum distance *d*_C=C_ for the photodimerization reaction is considered to be achieved. The presence of an external electric field suppressed the thermal fluctuations, resulting in a decrease in the reaction yield. These results indicate the possibility of controlling photodimerization reactions in the solid phase by an external field.

## Conclusion

To add ferroelectricity into a stilbene derivative, **C14SDA** with an alkyl amide chain was designed and its phase transition behavior, molecular assembly structure, dielectric properties, and photodimerization reaction were investigated in detail. **C14SDA** shows a sequential phase transition to the S1, S2, and S3 phases with an increasing temperature. In the S2 and S3 phases, a dynamic molecular assembly was formed in the partial melting of alkyl chains and thermal fluctuations of the intermolecular amide-type hydrogen bonds. In the temperature and frequency dependence of the dielectric constant, an increase in the dielectric constant in the low-frequency region was observed in the S2 and S3 phases, and hysteresis characteristic of ferroelectrics appeared in the electric field–polarization curve. In the S1 phase, the intermolecular —C=C— distance was 4.938 Å, and following Schmidt’s rule, no photodimerization reaction occurred. However, in the S2 and S3 phases, which are dynamic molecular assemblies, [2 + 2] photoaddition reactions proceeded. Particularly in the S3 phase, in addition to the formation of photodimerization product **C14CBDA**, the formation of photoisomerization product *cis*-**C14SDA** was also observed. Thermal fluctuations of the stilbene π-core in the high-temperature phase resulted in an intermolecular distance at which photodimerization reactions were possible. Furthermore, the application of an external electric field aligned the dipole moment direction, which modulated the —C=C— double bonds and resulted in a reduction of the photodimerization reaction yield. These results indicate the possibility of precisely controlling photoreactivity in the solid phase by further molecular design. The confirmation that control of dynamics in the solid phase is also extremely effective for photoreactivity is an important point.

## Supporting Information Available

Experimental section, crystal structure of **C4SDA**, TG charts, POM images, temperature-dependent IR spectra, ^1^H NMR spectra, PXRD, optimized molecular structure of photodimerized **C14CBDA**, and temperature- and photoirradiation-time-dependent conversion rate from **C14SDA** under the application of an electric field (PDF)

## References

[ref1] Baroncini M.; Groppi J.; Corra S.; Silvi S.; Credi A. Light-Responsive (Supra)Molecular Architectures: Recent Advances. Adv. Opt. Mater. 2019, 7, 1900392 10.1002/adom.201900392.

[ref2] Di Martino M.; Sessa L.; Diana R.; Piotto S.; Concilio S. Recent Progress in Photoresponsive Biomaterials. Molecules. 2023, 28, 3712 10.3390/molecules28093712. 37175122 PMC10180172

[ref3] Xu F.; Feringa B. L. Photoresponsive Supramolecular Polymers: From Light-Controlled Small Molecules to Smart Materials. Adv. Mater. 2023, 35, 2204413 10.1002/adma.202204413. 36239270

[ref4] Merino E.; Ribagorda M. Control over Molecular Motion Using the Cis–Trans Photoisomerization of the Azo Group. Beil. J. Org. Chem. 2012, 8, 1071–1090. 10.3762/bjoc.8.119. PMC3458724 23019434

[ref5] Cembran A.; Bernardi F.; Garavelli M.; Gagliardi L.; Orlandi G. On the Mechanism of the Cis–trans Isomerization in the Lowest Electronic States of Azobenzene: S0, S1, and T1. J. Am. Chem. Soc. 2004, 126, 3234–3243. 10.1021/ja038327y. 15012153

[ref6] Konieczkowska J.; Wasiak A.; Sobolewska A.; Bartkiewicz S.; Małecki J. G.; Schab-Balcerzak E. Kinetics of the Dark Cis–Trans Isomerization of Azobenzene and Azo Pyridine Derivatives in Ethanol and Chloroform Solutions. J. Photo. Photobio. A Chem. 2023, 444, 114979 10.1016/j.jphotochem.2023.114979.

[ref7] Merritt I. C. D.; Jacquemin D.; Vacher M. Cis → Trans Photoisomerisation of Azobenzene: A Fresh Theoretical Look. Phys. Chem. Chem. Phys. 2021, 23, 19155–19165. 10.1039/D1CP01873F. 34195720

[ref8] Han J.; Zheng Q.; Jin C.; Wang S.; Liu Y.; Zhao Y.; Zhu J. Comparative Study on Properties, Structural Changes, and Isomerization of Cis/Trans-Stilbene under High Pressure. J. Phys. Chem. C 2022, 126, 16859–16866. 10.1021/acs.jpcc.2c04865.

[ref9] Martinez Utrilla R.; Sastre R.; Catalina F.; Mateo J. L. Photosensitized Trans-Cis Isomerization of Stilbene in a Pure Polystyrene Matrix. J. Photochem. Photobiol. A. Chem. 1989, 46, 113–119. 10.1016/1010-6030(89)87037-6.

[ref10] Fuß W.; Kosmidis C.; Schmid W. E.; Trushin S. A. The Photochemical Cis–Trans Isomerization of Free Stilbene Molecules Follows a Hula-Twist Pathway. Angew. Chem., Int. Ed. 2004, 43, 4178–4182. 10.1002/anie.200454221. 15307081

[ref11] Latva-Mäenpää H.; Wufu R.; Mulat D.; Sarjala T.; Saranpää P.; Wähälä K. Stability and Photoisomerization of Stilbenes Isolated from the Bark of Norway Spruce Roots. Molecules 2021, 26, 1036 10.3390/molecules26041036. 33669268 PMC7920084

[ref12] Enkelmann V.; Wegner G.; Novak K.; Wagener K. B. Single-Crystal-to-Single-Crystal Photodimerization of Cinnamic Acid. J. Am. Chem. Soc. 1993, 115, 10390–10391. 10.1021/ja00075a077.

[ref13] Bushuyev O. S.; Singleton T. A.; Barrett C. J. Fast, Reversible, and General Photomechanical Motion in Single Crystals of Various Azo Compounds Using Visible Light. Adv. Mater. 2013, 25, 1796–1800. 10.1002/adma.201204831. 23335106

[ref14] Lee J.; Oh S.; Pyo J.; Kim J.-M.; Je J. H. A Light-Driven Supramolecular Nanowire Actuator. Nanoscale 2015, 7, 6457–6461. 10.1039/C5NR01118C. 25793329

[ref15] Saito K.; Ohnuma M.; Norikane Y. Negative Phototactic Behaviour of Crystals on A Glass Surface. Chem. Commun. 2019, 55, 9303–9306. 10.1039/C9CC03826D. 31309947

[ref16] Bushuyev O. S.; Tomberg A.; Friščić T.; Barrett C. J. Shaping Crystals with Light: Crystal-to-Crystal Isomerization and Photomechanical Effect in Fluorinated Azobenzenes. J. Am. Chem. Soc. 2013, 135, 12556–12559. 10.1021/ja4063019. 23924402

[ref17] Bushuyev O. S.; Tomberg A.; Vinden J. R.; Moitessier N.; Barrett C. J.; Friščić T. Azo···Phenyl Stacking: A Persistent Self-Assembly Motif Guides the Assembly of Fluorinated cis-Azobenzenes into Photo-mechanical Needle Crystals. Chem. Commun. 2016, 52, 2103–2106. 10.1039/C5CC08590J. 26691226

[ref18] Lai C.-Y.; Raj G.; Liepuoniute I.; Chiesa M.; Naumov P. Direct Observation of Photoinduced *trans–cis* Isomerization on Azobenzene Single Crystal. Cryst. Growth Des. 2017, 17, 3306–3312. 10.1021/acs.cgd.7b00288.

[ref19] Schmidt G. M. J. Topochemistry. Part III. The Crystal Chemistry of Some Trans-Cinnamic Acids. J. Chem. Soc. 1964, 1964, 2014–2021. 10.1039/jr9640002014.

[ref20] Fernandes M. A.; Levendis D. C. Photodimerization of the alpha’-polymorph of ortho-ethoxy-trans-cinnamic acid in the solid state. I. Monitoring the reaction at 293 K. Acta Cryst. Sect. B 2004, 60, 315–324. 10.1107/S0108768104007955. 15148435

[ref21] Schmidt G. M. J. Photodimerization in the Solid State. Pure Appl. Chem. 1971, 27, 647–678. 10.1351/pac197127040647.

[ref22] Yamada S.; Uematsu N.; Yamashita K. Role of Cation−π Interactions in the Photodimerization of Trans-4-Styrylpyridines. J. Am. Chem. Soc. 2007, 129, 12100–12101. 10.1021/ja074874y. 17880084

[ref23] Yu X.; Scheller D.; Rademacher O.; Wolff T. Selectivity in the Photodimerization of 6-Alkylcoumarins. J. Org. Chem. 2003, 68, 7386–7399. 10.1021/jo034627m. 12968891

[ref24] Toda F.; Tanaka K.; Kato M. Stereoselective Photodimerisation of Chalcones in the Molten State. J. Chem. Soc. Perkin. 1998, 1 (7), 1315–1318. 10.1039/a707380a.

[ref25] Chen X.-Y.; Chen H.; Đorđević L.; Guo Q.-H.; Wu H.; Wang Y.; Zhang L.; Jiao Y.; Cai K.; Chen H.; Stern C. L.; Stupp S. I.; Snurr R. Q.; Shen D.; Stoddart J. F. Selective Photodimerization in a Cyclodextrin Metal–Organic Framework. J. Am. Chem. Soc. 2021, 143, 9129–9139. 10.1021/jacs.1c03277. 34080831

[ref26] Hazra A.; Jain A.; Deenadayalan M. S.; Adalikwu S. A.; Maji T. K. Acetylene/Ethylene Separation and Solid-State Structural Transformation via [2 + 2] Cycloaddition Reactions in 3D Microporous Zn^II^ Metal–Organic Frameworks. Inorg. Chem. 2020, 59, 9055–9064. 10.1021/acs.inorgchem.0c00932. 32515587

[ref27] Park I.-H.; Lee E.; Lee S. S.; Vittal J. J. Chemical Patterning in Single Crystals of Metal–Organic Frameworks by [2 + 2] Cycloaddition Reaction. Angew. Chem., Int. Ed. 2019, 58, 14860–14864. 10.1002/anie.201904834. 31461210

[ref28] Peedikakkal A. M. P.; Peh C. S. Y.; Koh L. L.; Vittal J. J. Metal–Organic Frameworks Containing a Tetrapyridylcyclobutane Ligand Derived from Isomerization Reaction. Inorg. Chem. 2010, 49, 6775–6777. 10.1021/ic100853h. 20604545

[ref29] Kusaka S.; Kiyose A.; Sato H.; Hijikata Y.; Hori A.; Ma Y.; Matsuda R. Dynamic Topochemical Reaction Tuned by Guest Molecules in the Nanospace of a Metal–Organic Framework. J. Am. Chem. Soc. 2019, 141, 15742–15746. 10.1021/jacs.9b07682. 31550888

[ref30] Cao L.-H.; Xu X.-Q.; Tang X.-H.; Yang Y.; Liu J.; Yin Z.; Zang S.-Q.; Ma Y.-M. Controllable Strategy for Metal–Organic Framework Light-Driven [2 + 2] Cycloaddition Reactions via Solvent-Assisted Linker Exchange. Inorg. Chem. 2021, 60, 2117–2121. 10.1021/acs.inorgchem.0c02999. 33400525

[ref31] Hazra A.; Bonakala S.; Adalikwu S. A.; Balasubramanian S.; Maji T. K. Fluorocarbon-Functionalized Superhydrophobic Metal–Organic Framework: Enhanced CO_2_ Uptake via Photoinduced Postsynthetic Modification. Inorg. Chem. 2021, 60, 3823–3833. 10.1021/acs.inorgchem.0c03575. 33655749

[ref32] Sinnwell M. A.; Baltrusaitis J.; MacGillivray L. R. Combination of Argentophilic and Perfluorophenyl-Perfluorophenyl Interactions Supports a Head-to-Head [2 + 2] Photodimerization in the Solid State. Cryst. Grow. Des. 2015, 15, 538–541. 10.1021/cg501571u.

[ref33] Bera S.; Dutta B.; Mandal D.; Sinha C.; Mir M. H. A Dual Functional 2D MOF Exhibiting Rare Photosalient Effect as Well as Selective Pd(II) Sensing in Aqueous Medium. Inorg. Chem. 2022, 61, 13244–13249. 10.1021/acs.inorgchem.2c01740. 35972541

[ref34] Medishetty R.; Park I.-H.; Lee S. S.; Vittal J. J. Solid-State Polymerisation via [2 + 2] Cycloaddition Reaction Involving Coordination Polymers. Chem. Commun. 2016, 52, 3989–3990. 10.1039/C5CC08374E. 26687811

[ref35] Medishetty R.; Tandiana R.; Koh L. L.; Vittal J. J. Assembly of 3D Coordination Polymers from 2D Sheets by [2 + 2] Cycloaddition Reaction. Chem. - Eur. J. 2014, 20, 1231–1236. 10.1002/chem.201304246. 24382684

[ref36] Zhang Q.; Wang Y.; Braunstein P.; Lang J.-P. Construction of Olefinic Coordination Polymer Single Crystal Platforms: Precise Organic Synthesis, in situ Exploration of Reaction Mechanisms and Beyond. Chem. Soc. Rev. 2024, 53, 5227–5263. 10.1039/D3CS01050C. 38597808

[ref37] Cao C.; Xue X.-R.; Li Q.-Y.; Zhang M.-J.; Abrahams B. F.; Lang J.-P. Phase Transition-Promoted Rapid Photomechanical Motions of Single Crystals of a Triene Coordination Polymer. Angew. Chem., Int. Ed. 2023, 62, e202306048 10.1002/anie.202306048. 37186135

[ref38] Nakagawa M.; Kusaka S.; Kiyose A.; Nakajo T.; Iguchi H.; Mizuno M.; Matsuda R. Beyond the Conventional Limitation of Photocycloaddition Reaction in the Roomy Nanospace of a Metal–Organic Framework. J. Am. Chem. Soc. 2023, 145, 12059–12065. 10.1021/jacs.3c01225. 37222679

[ref39] Wang M.-F.; Mi Y.; Hu F.-L.; Niu Z.; Yin X.-H.; Huang Q.; Wang H.-F.; Lang J.-P. Coordination-Driven Stereospecific Control Strategy for Pure Cycloisomers in Solid-State Diene Photocycloaddition. J. Am. Chem. Soc. 2020, 142, 700–704. 10.1021/jacs.9b12358. 31870143

[ref40] Hu F.-L.; Mi Y.; Zhu C.; Abrahams B. F.; Braunstein P.; Lang J.-P. Stereoselective Solid-State Synthesis of Substituted Cyclobutanes Assisted by Pseudorotaxane-like MOFs. Angew. Chem., Int. Ed. 2018, 57, 12696–12701. 10.1002/anie.201806076. 30109769

[ref41] Gong W.-J.; Yang Z.-Y.; Hong Y.-X.; Liu D.; Niu Z.; Braunstein P.; Lang J.-P. Tetraolefin Stereospecific Photodimerization and Photopolymerization in Coordination Polymers. Sci. China Chem. 2022, 65, 1867–1872. 10.1007/s11426-022-1313-5.

[ref42] Yang Z.-Y.; Sang X.; Liu D.; Li Q.-Y.; Lang F.; Abrahams B. F.; Hou H.; Braunstein P.; Lang J.-P. Photopolymerization-Driven Macroscopic Mechanical Motions of a Composite Film Containing a Vinyl Coordination Polymer. Angew. Chem., Int. Ed. 2023, 62, e202302429 10.1002/anie.202302429. 36920791

[ref43] Zhang Y.; Zheng X.; Saito Y.; Takeda T.; Hoshino N.; Takahashi K.; Nakamura T.; Akutagawa T.; Noro S. Solution State-Like Reactivity in Super Flexible Crystalline Werner-type Metal Complex Solid. Angew. Chem., Int. Ed. 2024, 63, e202407924 10.1002/anie.202407924. 39092669

[ref44] Akutagawa T. Chemical Design and Physical Properties of Dynamic Molecular Assemblies. Bull. Chem. Soc. Jpn. 2021, 94, 1400–1420. 10.1246/bcsj.20200384.

[ref45] Akutagawa T.; Takeda T.; Hoshino N. Dynamics of Proton, Ion, Molecule, and Crystal Lattice in Functional Molecular Assemblies. Chem. Commun. 2021, 57, 8378–8401. 10.1039/D1CC01586A. 34369489

[ref46] Liu J.-C.; Ai Y.; Liu Q.; Zeng Y.-P.; Chen X.-G.; Lv H.-P.; Xiong R.-G.; Liao W.-Q. Solid–Liquid Crystal Biphasic Ferroelectrics with Tunable Biferroelectricity. Adv. Mater. 2023, 35, 2302436 10.1002/adma.202302436. 37202898

[ref47] Peng H.; Xu Z.-K.; Du Y.; Li P.-F.; Wang Z.-X.; Xiong R.-G.; Liao W.-Q. The First Enantiomeric Stereogenic Sulfur-Chiral OrganicFerroelectric Crystals. Angew. Chem., Int. Ed. 2023, 62, e202306732 10.1002/anie.202306732. 37272456

[ref48] Song X.-J.; Chen X.-G.; Liu J.-C.; Liu Q.; Zeng Y.-P.; Tang Y.-Y.; Li P.-F.; Xiong R.-G.; Liao W.-Q. Biferroelectricity of A Homochiral Organic Molecule in Both Solid Crystal and Liquid Crystal Phases. Nature Commun. 2022, 13, 6150 10.1038/s41467-022-33925-2. 36258026 PMC9579164

[ref49] Yuan G.; Kimura Y.; Kobayashi T.; Takeda T.; Hoshino N.; Akutagawa T. Ion Polarization-Assisted Hydrogen-Bonded Ferroelectrics in Liquid Crystalline Domain. Chem. Sci. 2021, 12, 13520–13529. 10.1039/D1SC03301H. 34777772 PMC8528045

[ref50] Liao W.-Q.; Zeng Y.-L.; Tang Y.-Y.; Xu Y.-Q.; Huang X.-Y.; Yu H.; Lv H.-P.; Chen X.-C.; Xiong R.-G. Dual Breaking of Molecular Orbitals and Spatial Symmetry in an Optically Controlled Ferroelectric. Adv. Mater. 2023, 35, 2305471 10.1002/adma.202305471. 37607776

[ref51] Takeda T.; Akutagawa T. Chemical Design of Organic Ferroelectrics Using Dynamics of Alkylamide Chains. Chem. Commun. 2022, 58, 11898–11912. 10.1039/D2CC04120K. 36200452

[ref52] Anetai H.; Wada Y.; Takeda T.; Hoshino N.; Yamamoto S.; Mitsuishi M.; Takenobu T.; Akutagawa T. Fluorescent Ferroelectrics of Hydrogen-Bonded Pyrene Derivatives. J. Phys. Chem. Lett. 2015, 6, 1813–1818. 10.1021/acs.jpclett.5b00703. 26263253

[ref53] Shishido Y.; Anetai H.; Takeda T.; Hoshino N.; Noro S.; Nakamura T.; Akutagawa T. Molecular Assembly and Ferroelectric Response of Benzenecarboxamides Bearing Multiple–CONHC_14_H_29_ Chains. J. Phys. Chem. C 2014, 118, 21204–21214. 10.1021/jp506035h.

[ref54] Mizoue R.; Kawana M.; Takeda T.; Hoshino N.; Akutagawa T. Ferroelectricity and Phase Change Memory of Bis(Tetradecylamide)-Substituted Benzene Derivatives. J. Phys. Chem. C 2023, 127, 1981–1991. 10.1021/acs.jpcc.2c07343.

[ref55] Kawana M.; Mizoue R.; Takeda T.; Hoshino N.; Akutagawa T. Simple Molecular Ferroelectrics: N,N′-Dialkyl-Terephthalamide Derivatives in the Solid Phase. J. Mater. Chem. C 2022, 10, 4208–4217. 10.1039/D1TC05001J.

[ref56] Wu J.; Zhu Q.; Takeda T.; Hoshino N.; Akutagawa T. Ferroelectricity of Hydrogen-Bonded Azobenzene Derivatives. ACS Appl. Electron. Mater. 2021, 3, 3521–3529. 10.1021/acsaelm.1c00462.

[ref57] Gorbunov A. V.; Meng X.; Urbanaviciute I.; Putzeys T.; Wübbenhorst M.; Sijbesma R. P.; Kemerink M. Polarization Loss in the Organic Ferroelectric Trialkylbenzene-1,3,5-Tricarboxamide (BTA). Phys. Chem. Chem. Phys. 2017, 19, 3192–3200. 10.1039/C6CP08015D. 28083589

[ref58] Gorbunov A. V.; Putzeys T.; Urbanavičiu̅tė I.; Janssen R. A. J.; Wübbenhorst M.; Sijbesma R. P.; Kemerink M. True Ferroelectric Switching in Thin Films of Trialkylbenzene-1,3,5-Tricarboxamide (BTA). Phys. Chem. Chem. Phys. 2016, 18, 23663–23672. 10.1039/C6CP03835B. 27510767

[ref59] Fitié C. F. C.; Roelofs W. S. C.; Magusin P. C. M. M.; Wübbenhorst M.; Kemerink M.; Sijbesma R. P. Polar Switching in Trialkylbenzene-1,3,5-Tricarboxamides. J. Phys. Chem. B 2012, 116, 3928–3937. 10.1021/jp300008f. 22397532

[ref60] Fitié C. F. C.; Tomatsu I.; Byelov D.; de Jeu W. H.; Sijbesma R. P. Nanostructured Materials through Orthogonal Self-Assembly in a Columnar Liquid Crystal. Chem. Mater. 2008, 20, 2394–2404. 10.1021/cm703508t.

[ref61] Fitié C. F. C.; Roelofs W. S. C.; Kemerink M.; Sijbesma R. P. Remnant Polarization in Thin Films from a Columnar Liquid Crystal. J. Am. Chem. Soc. 2010, 132, 6892–6893. 10.1021/ja101734g. 20441217

[ref62] Sambe K.; Takeda T.; Hoshino N.; Matsuda W.; Miura R.; Tsujita K.; Maruyama S.; Yamamoto S.; Seki S.; Matsumoto Y.; Akutagawa T. Ferroelectric Organic Semiconductor: BTBT Bearing Hydrogen-Bonding–CONHC_14_H_29_ Chain. ACS Appl. Mater. Interfaces 2023, 15, 58711–58722. 10.1021/acsami.3c14476. 38055344

[ref63] Sambe K.; Takeda T.; Hoshino N.; Matsuda W.; Miura R.; Tsujita K.; Maruyama S.; Yamamoto S.; Seki S.; Matsumoto Y.; Akutagawa T. Carrier Transport Switching of Ferroelectric BTBT Derivative. J. Am. Chem. Soc. 2024, 146, 8557–8566. 10.1021/jacs.4c00514. 38484118

[ref64] Mizoue R.; Takeda T.; Dekura S.; Kato M.; Fukui T.; Shoji Y.; Fukushima T.; Yamane S.; Suzuki Y.; Kawamata J.; Akutagawa T. Ferroelectricity of Alkylamide-Substituted Triptycene Derivative. J. Mater. Chem. C 2024, 12, 5578–5586. 10.1039/D3TC04752K.

[ref65] Wu J.; Takeda T.; Hoshino N.; Suzuki Y.; Kawamata J.; Akutagawa A. Ferroelectricity of a Tetraphenylporphyrin Derivative Bearing–CONHC_14_H_29_ Chains at 500 K. J. Phys. Chem. C 2019, 123, 22439–22446. 10.1021/acs.jpcc.9b03866.

[ref66] Anetai H.; Sambe K.; Takeda T.; Hoshino N.; Akutagawa T. Nanoscale Effects in One-Dimensional Columnar Supramolecular Ferroelectrics. Chem.—Eur. J. 2019, 25, 11233–11239. 10.1002/chem.201902544. 31250470

[ref67] Zhang Y.; Takeda T.; Hoshino N.; Akutagawa T. Crystal Design of Photodimerization and Proton Dynamics in Stilbene Dicarboxylate Salts. Cryst. Grow. Des. 2023, 23, 9121–9131. 10.1021/acs.cgd.3c01212.

[ref68] Veerakanellore G. B.; Captain B.; Ramamurthy V. Solid-State Photochemistry of cis-Cinnamic Acids: A Competition Between [2 + 2] Addition and cis–trans Isomerization. CrystEngComm 2016, 18, 4708–4712. 10.1039/C6CE00682E.

[ref69] MacGillivray L. R.; Reid J. L.; Ripmeester J. A. Supramolecular Control of Reactivity in the Solid State Using Linear Molecular Templates. J. Am. Chem. Soc. 2000, 122, 7817–7818. 10.1021/ja001239i.

[ref70] Crystal Structure: Single Crystal Structure Analysis Software, ver. 4.30; Rigaku Corporation and Molecular Structure Corporation: 2018.

[ref71] Sheldrick G. M. SHELX2014 Programs for Crystal Structure Analysis; Universitat Göttingen: Göttingen, Germany, 2014.

