# Supplementary material for: Photodimerization of Ferroelectric N,N′-Ditetradecyl-stilbenediamide Derivative
Source: J Am Chem Soc. 2025 Feb 24;147(9):7983–92. doi: 10.1021/jacs.5c00346 (PMC11887429; doi:10.1021/jacs.5c00346)
Supplement: Supplementary file 1 — ja5c00346_si_001.pdf [file ja5c00346_si_001.pdf]

Photodimerization of Ferroelectric *N*, *N*'-Ditetradecyl-stilbenediamide Derivative

Yunya Zhang,[a] Takashi Takeda,[a, b, c] and Tomoyuki Akutagawa[a, b]*

[a] Graduate School of Engineering, Tohoku University, Sendai 980-8579, Japan

[b] Institute of Multidisciplinary Research for Advanced Materials (IMRAM), Tohoku University, 2-1-1 Katahira, Aoba-ku, Sendai 980-8577, Japan.

[c] Faculty of Science, Shinshu University, Matsumoto 3-1-1, 390-8621, Japan.

Phone: +81-22-217-5653

Fax: +81-22-217-5655

E-mail akutagawa@tohoku.ac.jp

Contents

1. Experimental section (Figure S1).

2. Crystal Data, Data Collection, and Reduction Parameters of **C4SDA** (Table S1).

3. TG charts (Figure S2).

4. POM images of **C14SDA** (Figure S3).

5. Crystallographic and atomic numbering scheme in **C4SDA** (Figure S4).

6. Temperature-dependent PXRD data (Table S2).

7. Temperature-dependent IR spectra on KBr pellet of **C14SDA** (Figure S5).

8. DSC and chart and ^{1}H NMR spectra of **C4SDA** before and after photoirradiation (Figure S6).

9. ^{1}H NMR spectrum of **C14SDA** in $CDCl_3$ (Figure S7).

10. ^{1}H NMR spectrum of **C14SDA** after photoirradiation at 470 K (Figure S8).

11. ^{1}H NMR spectrum of **C14CBDA** in $CDCl_3$ (Figure S9).

12. ^{1}H-NMR spectra of photo-irradiation products of **C14SDA** at each phase (Figure S10-S14).

13. Photo-irradiation time- and temperature-dependent ^{1}H-NMR spectra of photoreaction compounds (Figures S15-S34).

14. Powder diffraction of **C14CBDA** (Figure S35).

15. TG chart of **C14CBDA**•0.5($CHCl_3$) (Figure S36).

16. Optimized molecular structure of photo-dimerized **C14CBDA** (Figure S37).

17. Temperature-dependent conversion rate of **C4SDA** (Figure S38)

18. Temperature- and photo-irradiation time (t) dependent conversion rate from **C14SDA**. The data under the application of an electric field were added (Figure S39).

Experimental Section

Physical measurements. Elemental analyses were performed on a Microcoder JM10 at the Elementary Analysis Laboratory, Institute of Multidisciplinary Research for Advanced Materials, Tohoku University. UV-vis-NIR and IR spectra were recorded on PerkinElmer Lambda 750 and Thermo Fisher Scientific Nicolet 6700 FT-IR spectrophotometers, respectively. Thermogravimetric (TG) differential thermal analysis and differential scanning calorimetry (DSC) were conducted using a Rigaku Thermo plus TG8120 thermal analysis station and Mettler DSC1-T with an Al_2O_3 reference and a heating and cooling rate of 5 K min^{-1} under nitrogen. Temperature-dependent dielectric constants were measured using the two-probe AC impedance method from 100 Hz to 1 MHz (Hewlett-Packard, HP4194A) and the temperature controller of a Linkam LTS-E350 system. The electrical contacts were prepared using gold paste (Tokuriki 8560) to attach the 10-μm ϕ gold wires to a single crystal.

Preparation of C4DSA and C14SDA. Commercially available reagents and solvents were used as received. A suspension of 4,4'-stilbenedicarboxylic acid (2 g, 7.45 mmol) in thionyl chloride (50 mL) with a catalytic amount of dry dimethylformamide (DMF) (2.5 mL) was refluxed for 24 hours at 333 K. Although the dicarboxylic acid did not dissolve initially, it was directly converted into the diacid chloride, which dissolved around 330 K. The resulting yellow crystalline product of stilbene-4,4'-dicarbonyl dichloride was isolated using a rotary evaporator, followed by vacuum drying for 6 hours. Due to its sensitivity to air, the product was used immediately for the next reaction without further purification. The obtained product was reacted with tetradecylamine (3.5 g, 18.63 mmol, 2.2 equivalents) in THF (150 mL) and triethylamine (2.5 mL). The mixture was refluxed for 24 hours at 333 K. The solid product was collected by filtration, washed with ethanol

(200 mL), and recrystallized from DMF (yield was 43%). Elemental analysis: Calculated for $C_{40}H_{70}N_2O_2$: C, 80.19; H, 10.71; N, 4.25%. Found: C, 80.30; H, 10.60; N, 4.36%.

Stilbene-4,4'-dicarbonyl dichloride was reacted with butylamine (1.6 mL, 16.39 mmol, 2.2 equivalents) in THF (100 mL) and triethylamine (2.5 mL). After refluxing for 24 hours at 333 K, the reaction product was filtered, washed with ethanol (100 mL), and yielded at 44%. Elemental analysis: Calculated for $C_{24}H_{30}N_2O_2$: C, 76.16; H, 7.99; N, 7.40%. Found: C, 76.12; H, 8.10; N, 7.50%.

Crystal structural determination. Crystallographic data were collected using a Rigaku RAPID-II diffractometer equipped with a rotating anode fitted with a multilayer confocal optic and using Cu Kα (λ = 1.54187 Å) radiation from a graphite monochromator. Structural refinements were performed using the full-matrix least-squares method on F^2. Calculations were performed using Crystal Structure software packages.[1] All the parameters, except for those of the hydrogen atoms, were refined using anisotropic temperature factors. Table S1 summarizes crystal data and parameters of **C4SDA**.

Table S1. **Crystal Data, Data Collection, and Reduction Parameters of C4SDA.**

**Crystal**	**C4SDA**
Chemical formula	$C_{24}H_{30}N_2O_2$
Formula weight	378.51
T, K	173
Space group	*P*-1 (#2)
a, Å	5.1735(3)
b, Å	8.1516(6)
c, Å	24.1289(17)
α, deg	96.901(7)
β, deg	93.389(7)
γ, deg	92.951(7)
V, $Å^3$	1006.68(12)
Z	2
D_{calc}, g·cm^{-3}	1.249
μ, cm^{-1}	6.229
Reflections measured	11011
Independent reflections	3585
Reflections used	3585
R_{int}	0.1073
R_1 [a]	0.0804
R_{all}	0.1327
$R_w(F_2)$ [a]	0.1833
GOF	1.041
CCDC	2361629

[a] $R_1 = \Sigma||F_o| - |F_c|| / \Sigma |F_o|$ and $R_w = (\Sigma\omega(|F_o| - |F_c|)2 / \Sigma\omega F_o^2)^{1/2}$.

Solid state photoreaction. Solid state photoreaction for the powder sample of **C14SDA** was conducted using a photoirradiation system assembled on an optical platform (Sigma Koki), mercury-lamp (Hamamatsu L2570 200W), xenon lamp power supply (Hamamatsu model C7535, output model 7.5 A) and starter unit (Hamamatsu model C4251) which was passed through the lens set (CHUO-SEKI) and sliver light filter (Asahi spectra, 365nm) to use the irradiation wavelength at 365 nm. Temperature control was meticulously managed by Linkam LTS-E350 system, which also provided an inert atmosphere for the reaction. The irradiation light power around the sample was confirmed at 4 W cm^{-2} using a UV power meter (Hamamatsu C6080-03). A powdered sample of **C14SDA** was sandwiched by the two quartz glass plates with a 1 mm deep slot. The result of photoreaction was evaluated by ^{1}H NMR spectra in $CDCl_3$ using Bruker Avance III 500 NMR spectrometer and Bruker Avance III 400 NMR spectrometer. Chemical shifts (δ) are expressed in ppm relative to tetramethylsilane (0.00 ppm) as an internal standard. The photodimerization reaction under an electric field in the ferroelectric phase was carried out by irradiation with 4 mW cm^{-2} light at 450 K for 90 min. The cell thicknesses of the sandwich cells used for the light irradiation were 100 and 300 mm and the applied voltages were 20 and 40 V.

Isolation of photodimerized C14CBDA. **C14CBDA** was isolated from the reaction products after the UV irradiation to powdered **C14SDA**. The intensity of the UV light, measured through the glass window of the Linkam, was fixed at 4 mW cm^{-2} under a nitrogen atmosphere at 450 K. The reaction products were completely dissolved in $CHCl_3$ at 333 K and immediately filtered through a PTFE syringe filter (0.45 µm). The filtrate solution was left in a dark place overnight. The evaporation of $CHCl_3$ yielded the powder **C14CBDA**. Elemental analysis of (**C14CBDA**)0.5$CHCl_3$: Calculated for $C_{88}H_{140}N_4O_4$0.5($CHCl_3$): C, 77.10; H, 10.19; N, 4.04%.

Found: C, 77.36; H, 10.45; N, 4.04%. HRMS (FAB$^+$) Calc. for $C_{88}H_{140}N_4O_4$ [(M+H)$^+$]: 1319.0857, found 1318.0953.

a)

b)

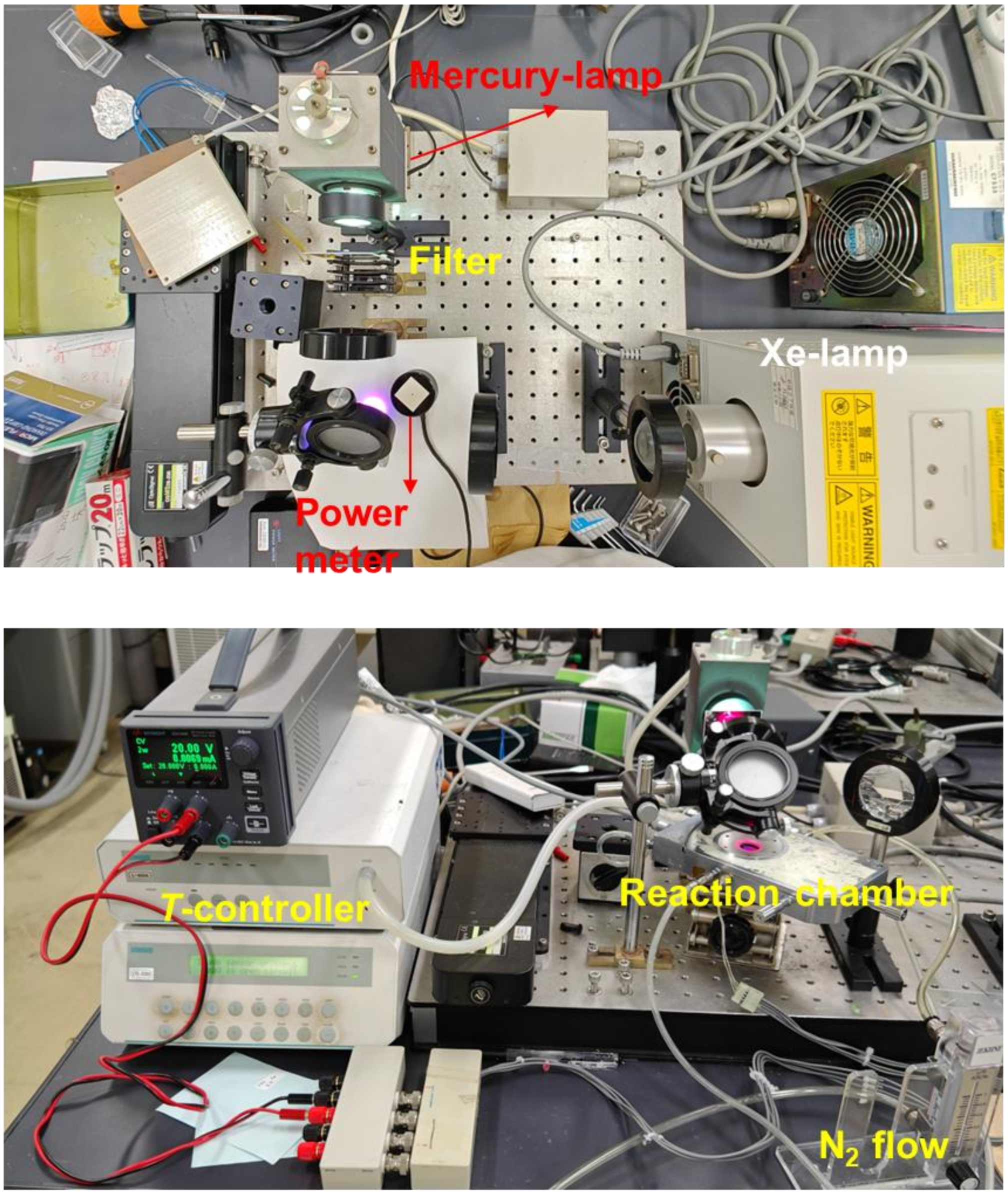

Figure S1. Photograph of a hand-made light irradiation instrument used in a light irradiation experiment of **C14SDA**. a) Photoirradiation unit and b) temperature control unit.

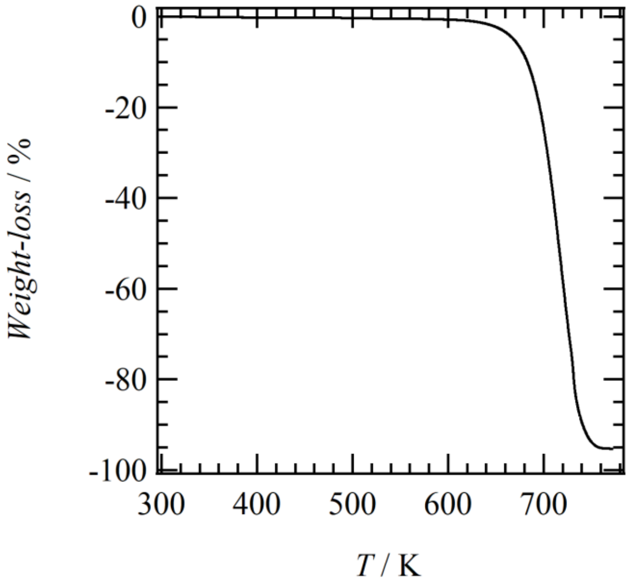

Figure S2. TG charts of **C14SDA**.

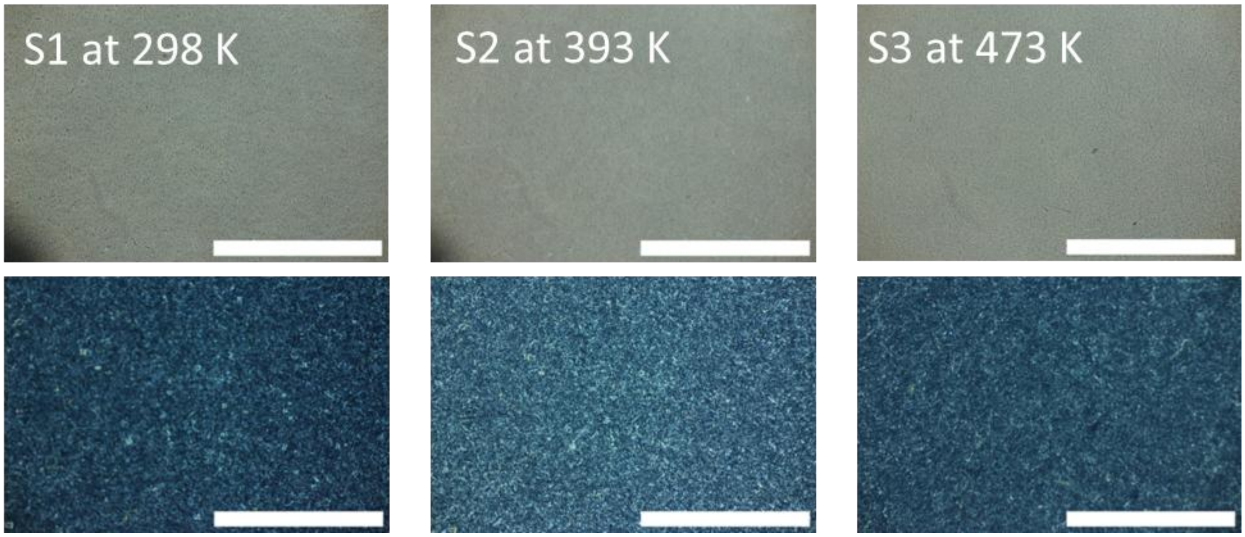

Figure S3. POM images of **C14SDA** at S1, S2, and S3 phases. The upper and lower images are without and with a cross-Nicole optical arrangement. Scale bars are 500 µm.

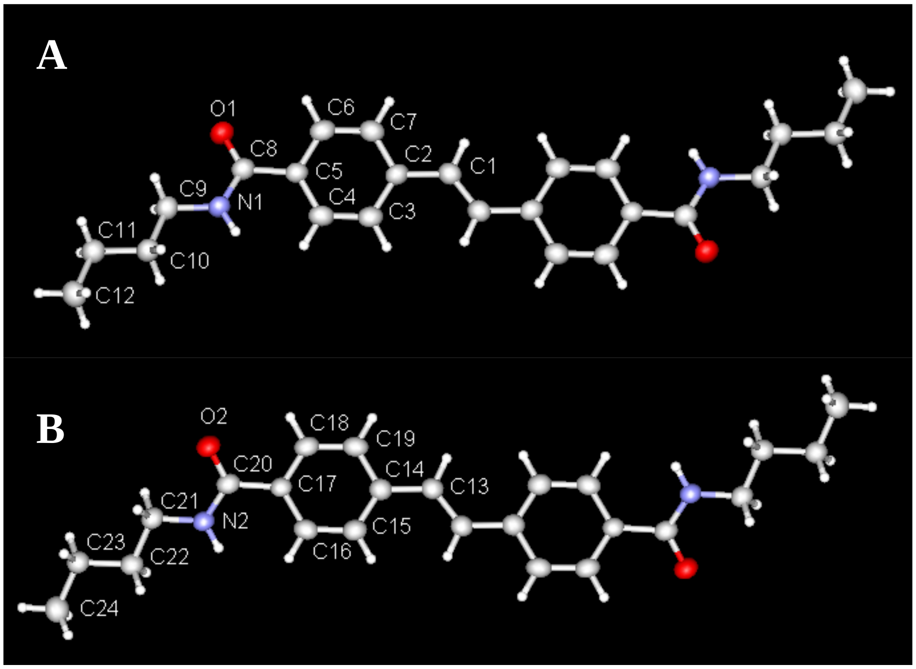

Figure S4. Crystallographic independent **C4SDA** molecules of **A** and **B**. The inversion center exists on each molecule.

Table S2. The relationship between 2θ and *d*-spacing in the temperature-dependent PXRD pattern of **C14SDA**.

T	2θ,	deg			*d*	Å		
300	7.55,	11.5			11.71	7.80		
403	3.62	5.37	7.20	10.78	24.41	16.46	12.28	8.21
443	3.62	5.42	7.29	10.92	24.41	16.31	12.13	7.10
473	7.72	11.53			11.45	7.68		
410	7.44	11.14			11.88	7.94		
388	7.31	10.92			12.09	8.10		
300	7.59	11.27			11.64	7.85		

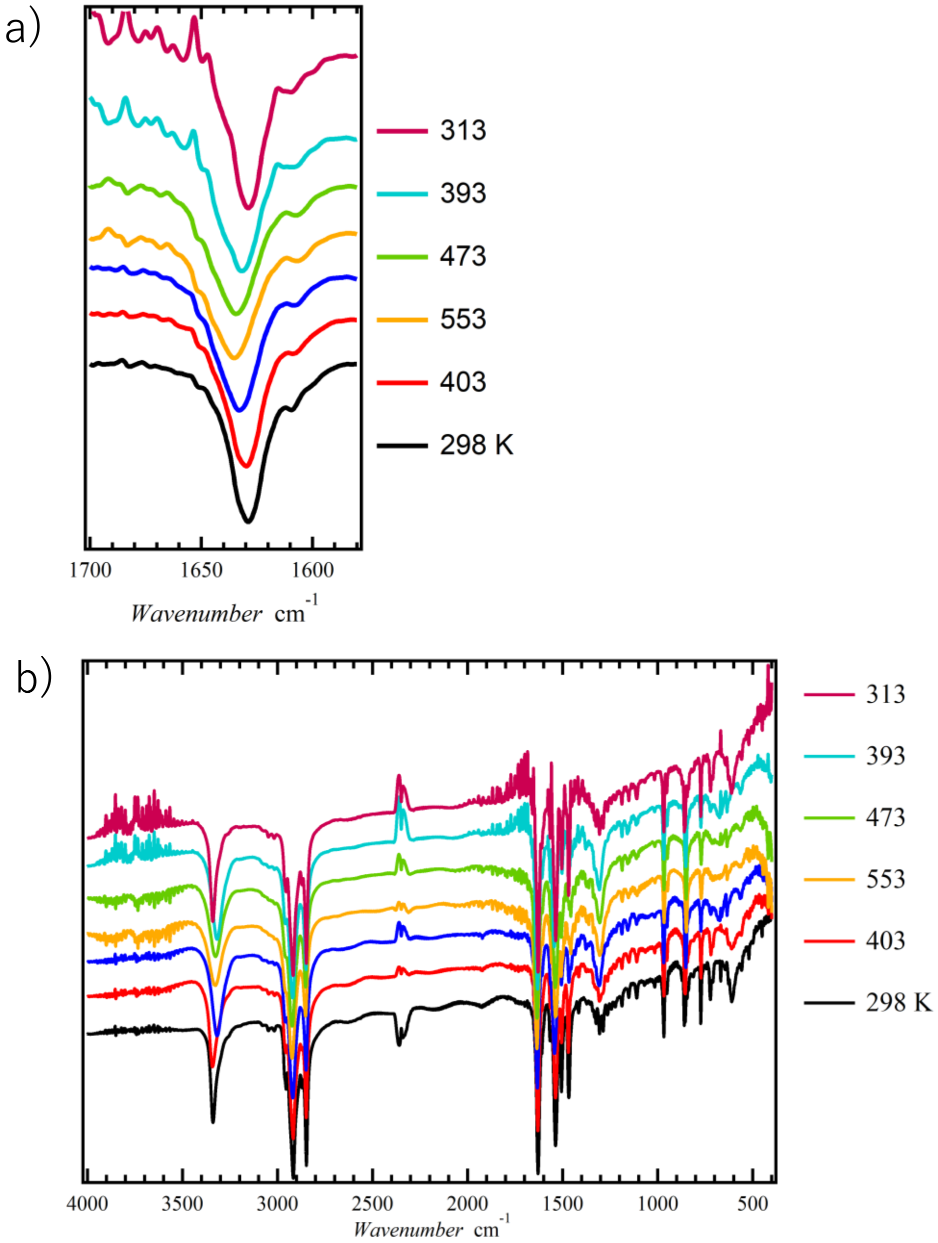

Figure S5. Temperature-dependent IR spectra of **C14SDA** on KBr pellet. Energy ranges at a) 1700-1580 cm^{-1} and 4000-400 cm^{-1}.

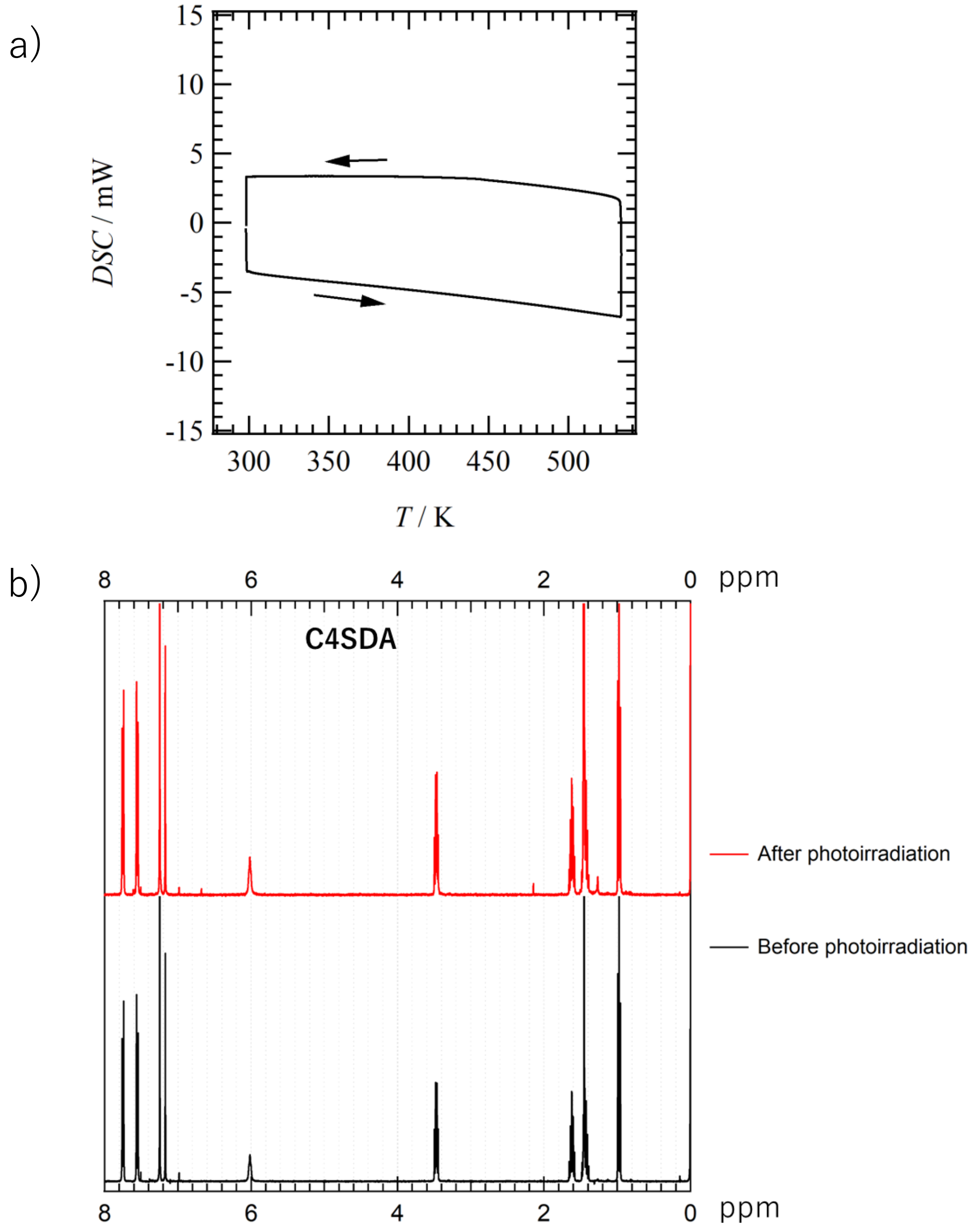

Figure S6. Thermal property and photodimerization reaction of **C4SDA**. a) DSC chart of **C4SDA** without phase transition and b) ^{1}H NMR spectra of **C4SDA** before and after photoirradiation at 298 K (365 nm, 4 mW cm^{-2}, and 60 min).

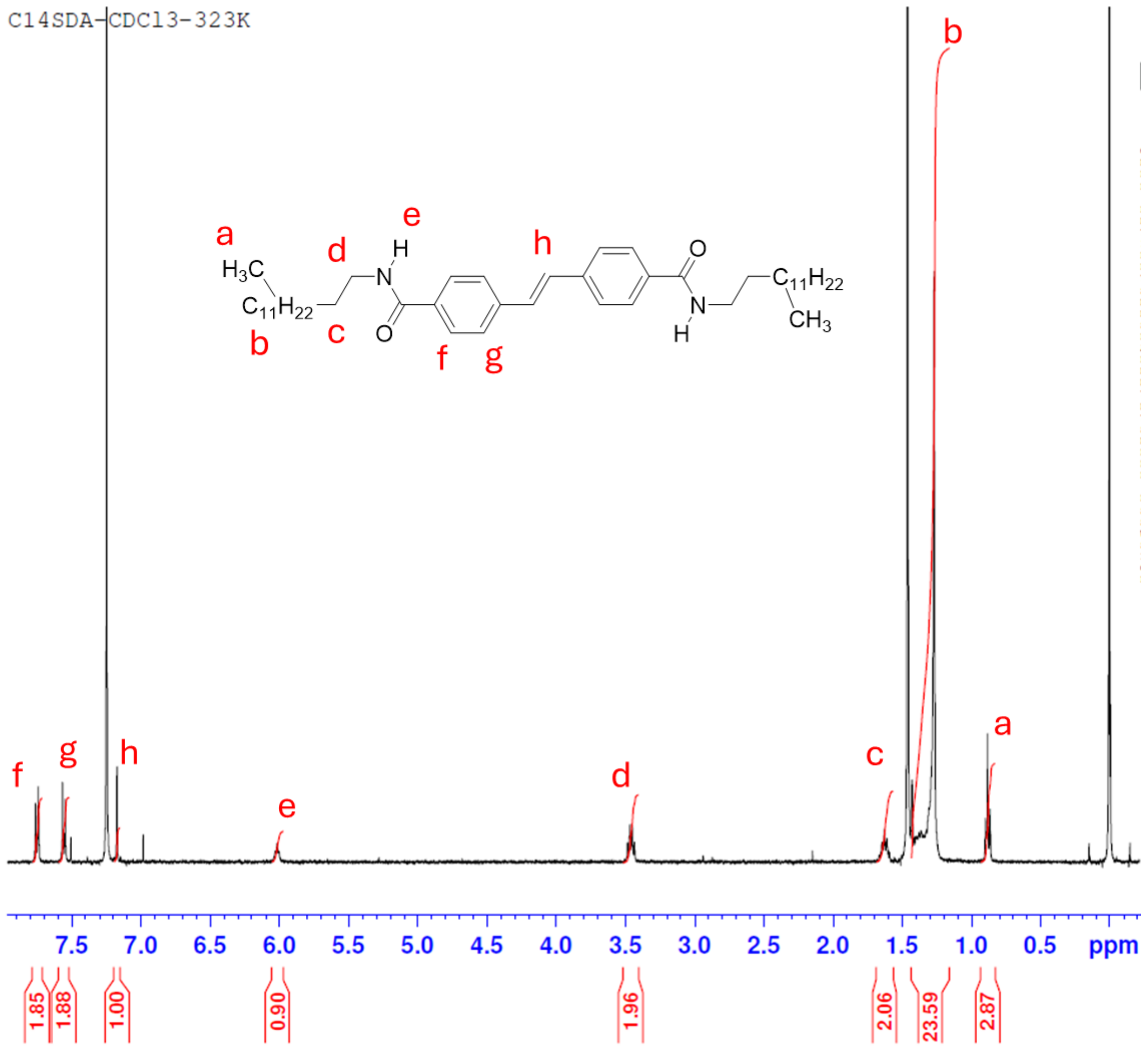

Figure S7. ^{1}H NMR spectrum of **C14SDA** in $CDCl_3$. Two strong signals at 1.6 and 7.3 ppm were due to H_2O and $CHCl_3$.

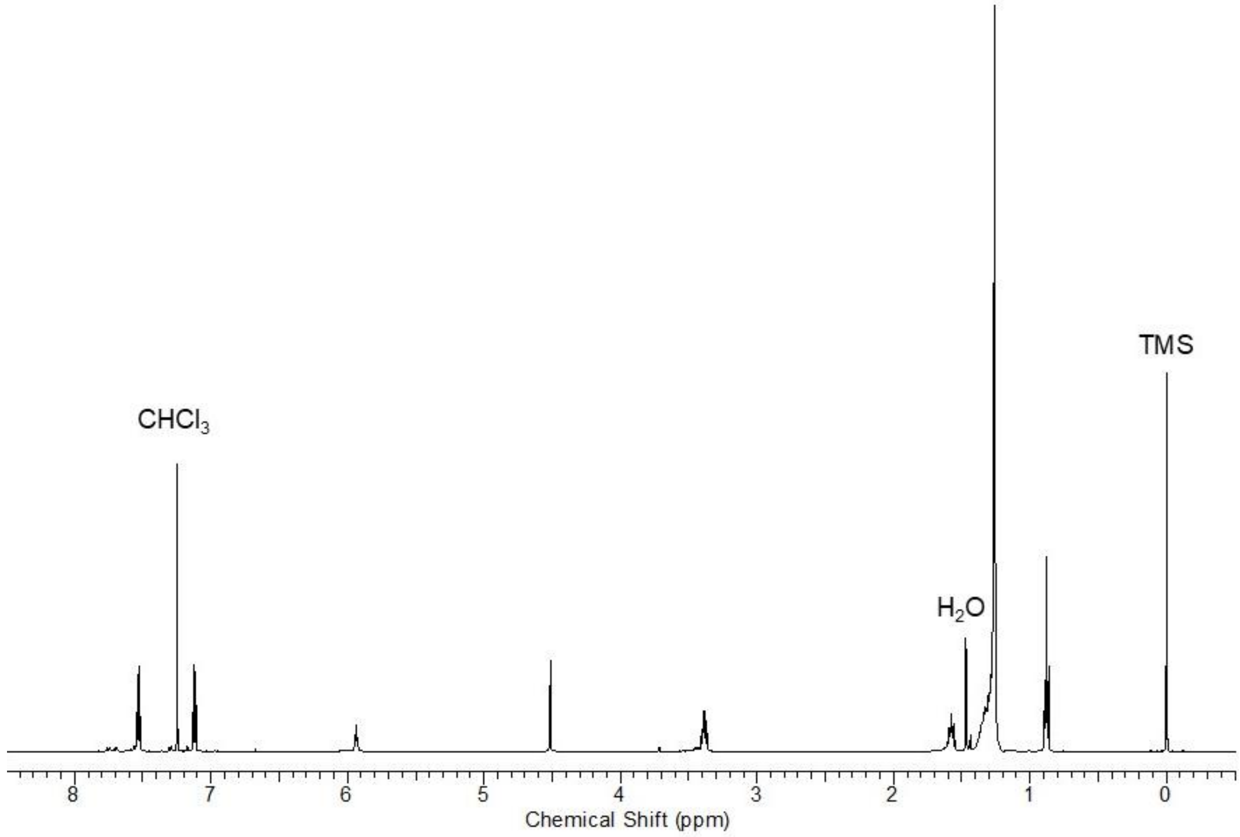

Figure S8. ^{1}H NMR spectrum of **C14SDA** after photoirradiation at 470 K for 120 min. The magnified spectrum of aromatic region stacked with pure **C14SDA** and **C14CBDA** and their assignment can be found in Figure 7b. The signal that appeared at 3.7 ppm was due to unidentified side product generated during the photoirradiation. Other signals were derived from **C14SDA**, *cis*-**C14SDA** and **C14CBDA**, except for those from $CHCl_3$, H_2O and TMS.

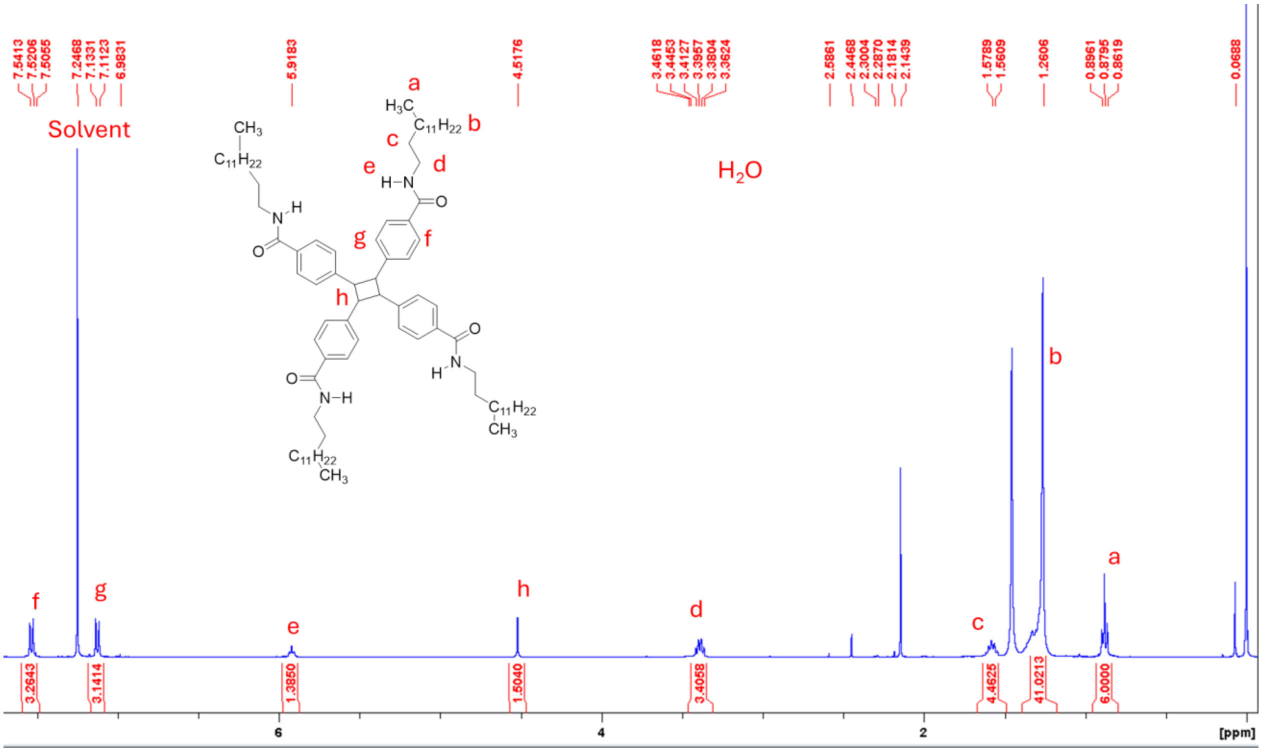

Figure S9. ^{1}H NMR spectrum of **C14CBDA** in $CDCl_3$. Two strong signals at 1.6 and 7.3 ppm were due to H_2O and $CHCl_3$.

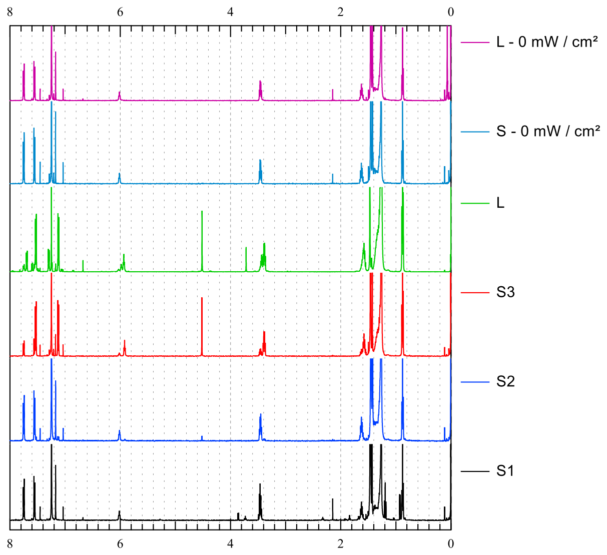

Figure S10. ^{1}H-NMR spectra of photo-irradiation (power 4 mW cm^{-2} and time 30 min) products of **C14SDA** at S1 (black), S2 (blue), S3 (red), and L (green) phases. To confirm the thermal dimerization reaction, the control experiments without a photo-irradiation were evaluated at S3 (S3-0. blue) and L (L-0. violet).

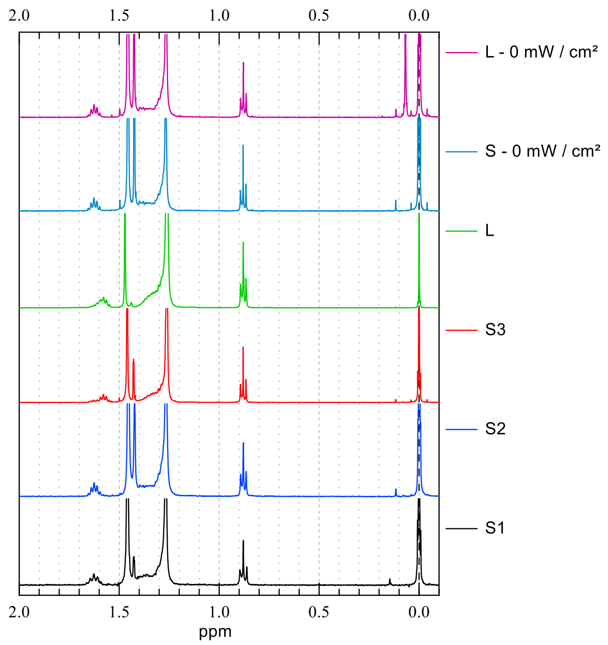

Figure S11. Expanded ^{1}H-NMR spectra of Figure S10 from 0.0 to 2.0 ppm.

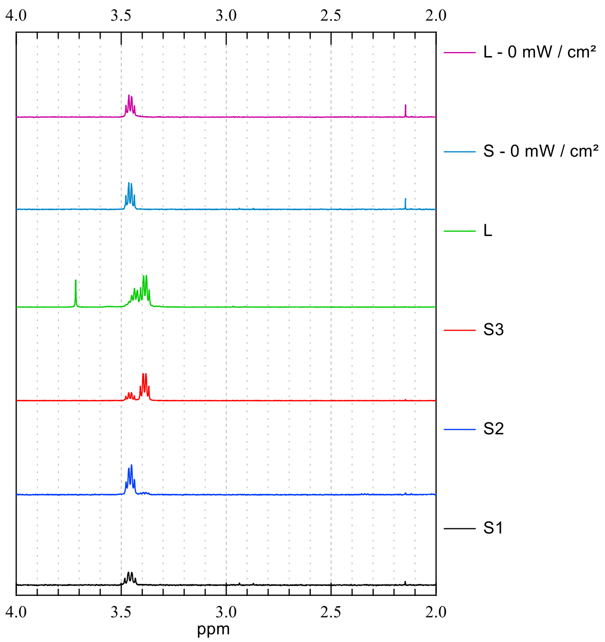

Figure S12. Expanded ^{1}H-NMR spectra of Figure S10 from 2.0 to 4.0 ppm.

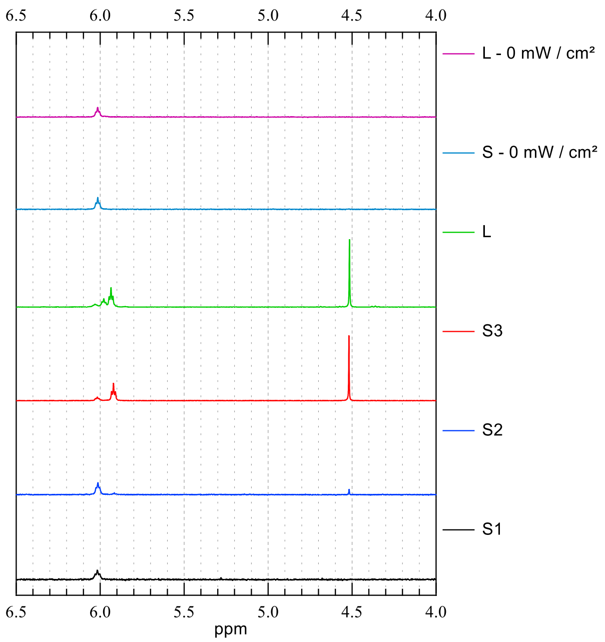

Figure S13. Expanded ^{1}H-NMR spectra of Figure S10 from 4.0 to 6.5 ppm.

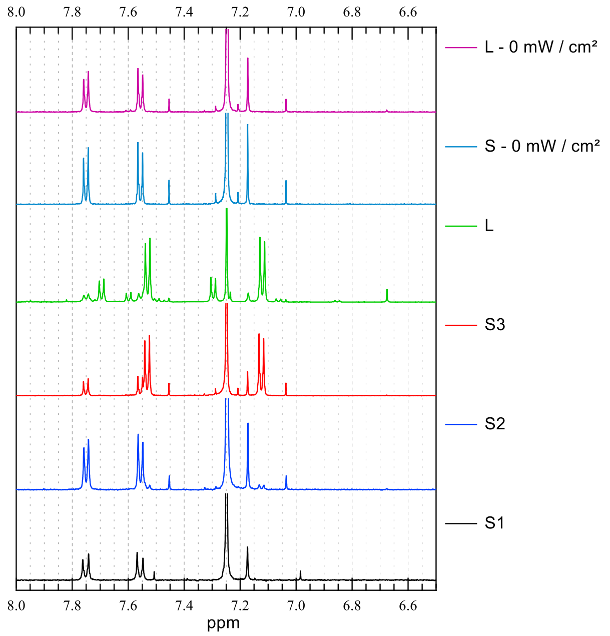

Figure S14. Expanded ^{1}H-NMR spectra of Figure S10 from 6.5 to 8.0 ppm.

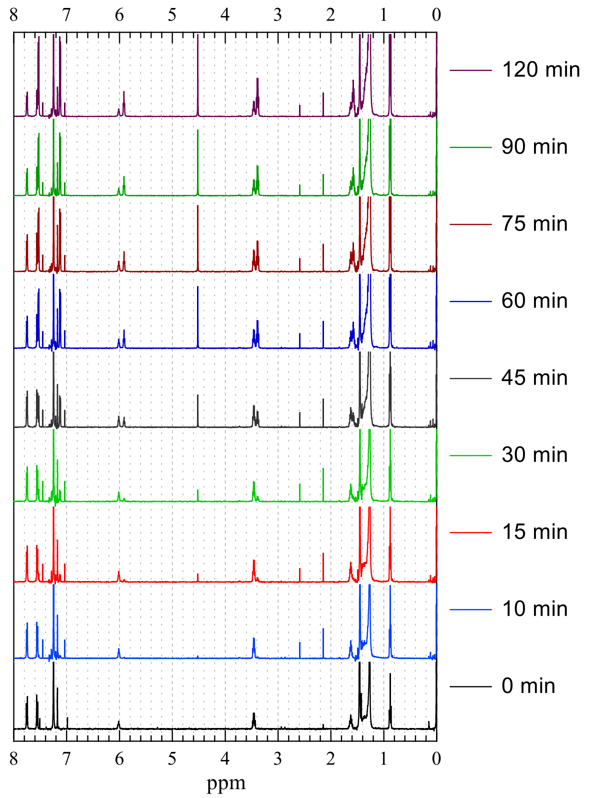

Figure S15. Photo-irradiation time-dependent ^{1}H-NMR spectra of **C14SDA** at 450 K.

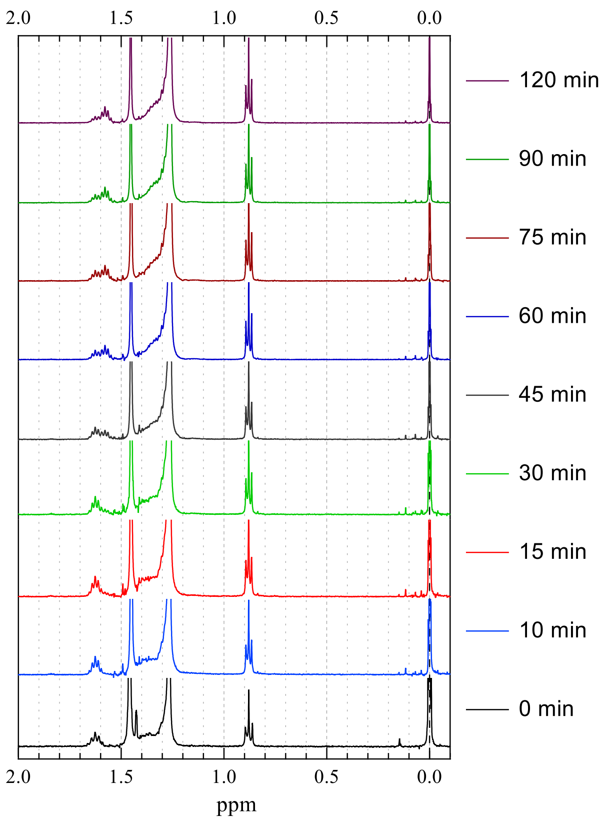

Figure S16. Expanded ^{1}H-NMR spectra of Figure S15 from 0.0 to 2.0 ppm.

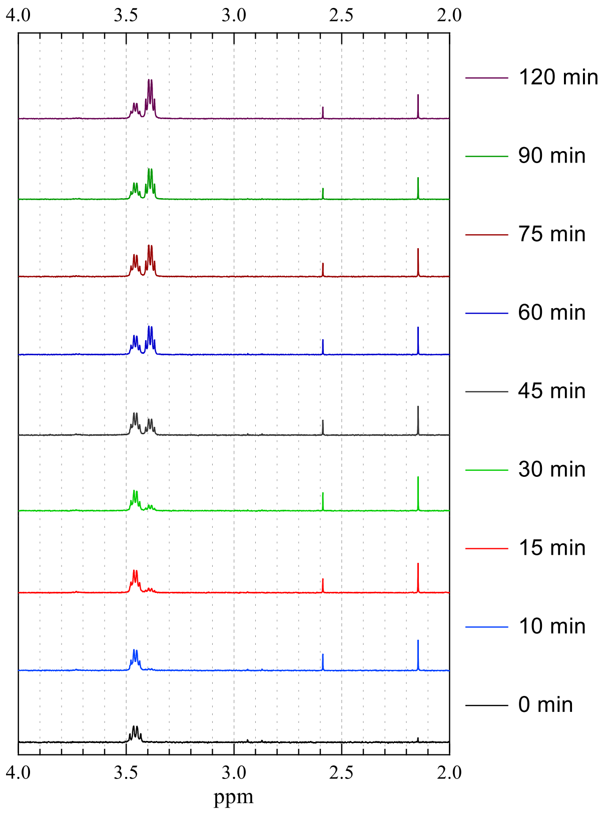

Figure S17. Expanded ^{1}H-NMR spectra of Figure S15 from 2.0 to 4.0 ppm.

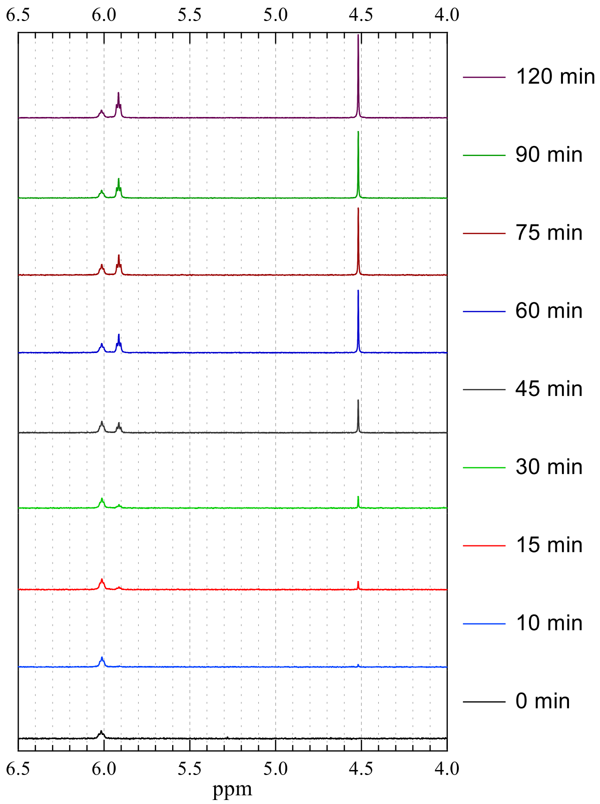

Figure S18. Expanded ^{1}H-NMR spectra of Figure S15 from 4.0 to 6.5 ppm.

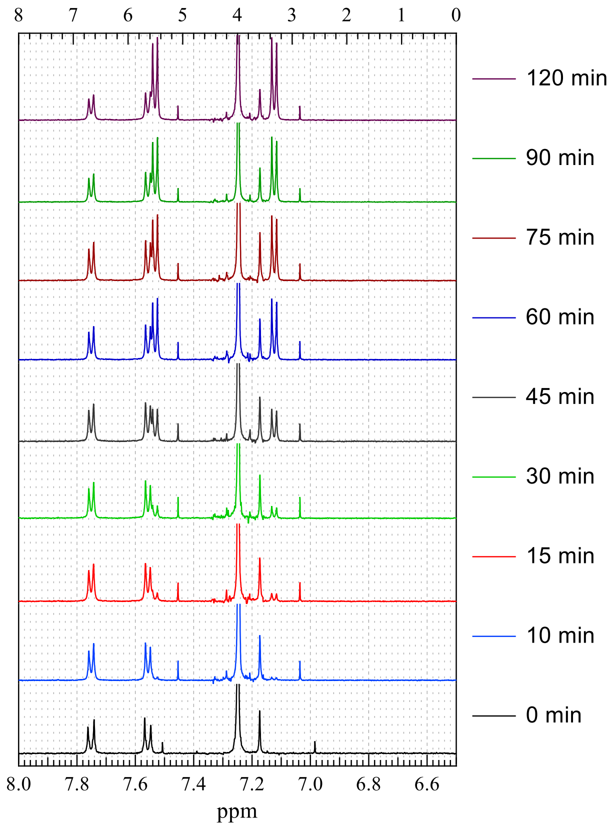

Figure S19. Expanded ^{1}H-NMR spectra of Figure S15 from 6.5 to 8.0 ppm.

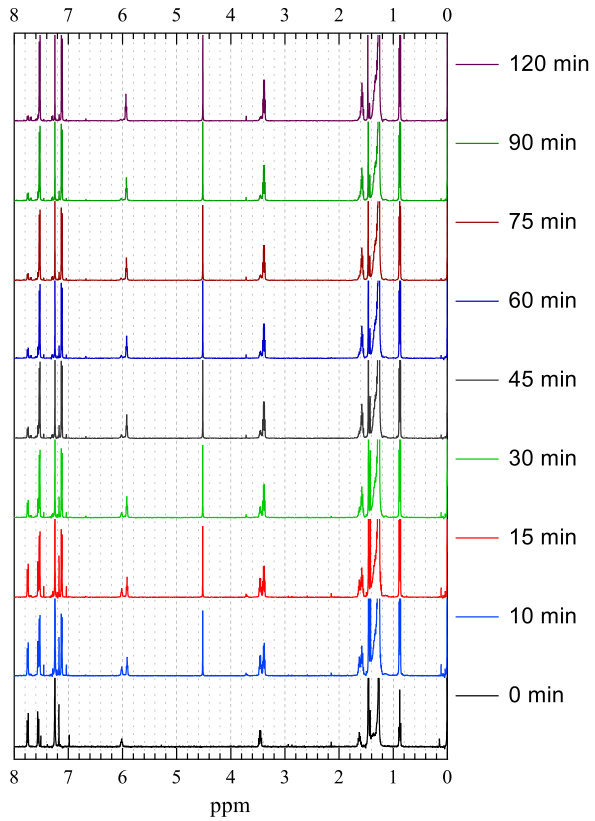

Figure S20. Photo-irradiation time-dependent ^{1}H-NMR spectra of **C14SDA** at 470 K.

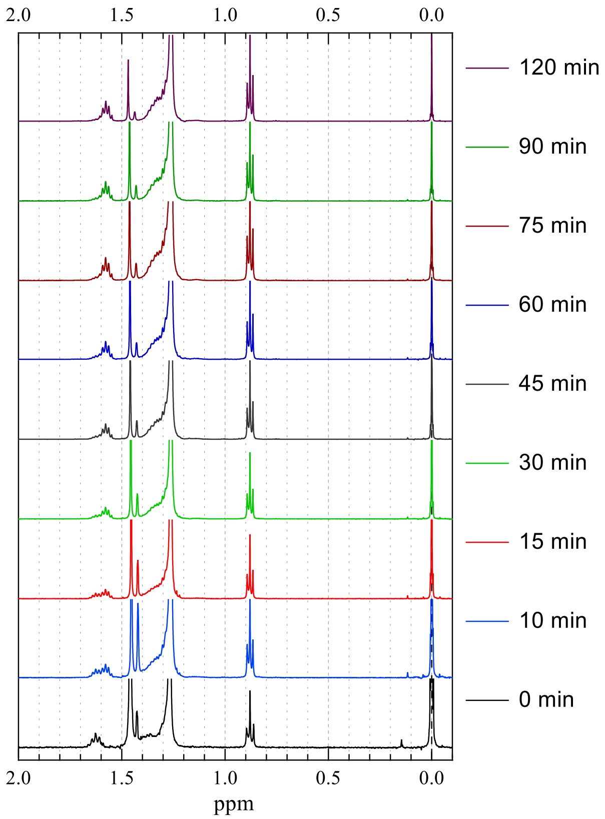

Figure S21. Expanded ^{1}H-NMR spectra of Figure S20 from 0.0 to 2.0 ppm.

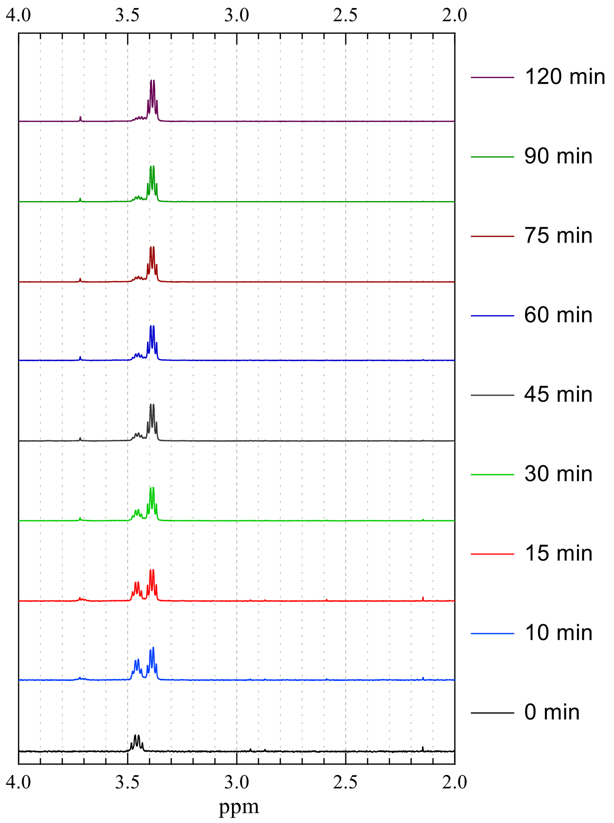

Figure S22. Expanded ^{1}H-NMR spectra of Figure S20 from 2.0 to 4.0 ppm.

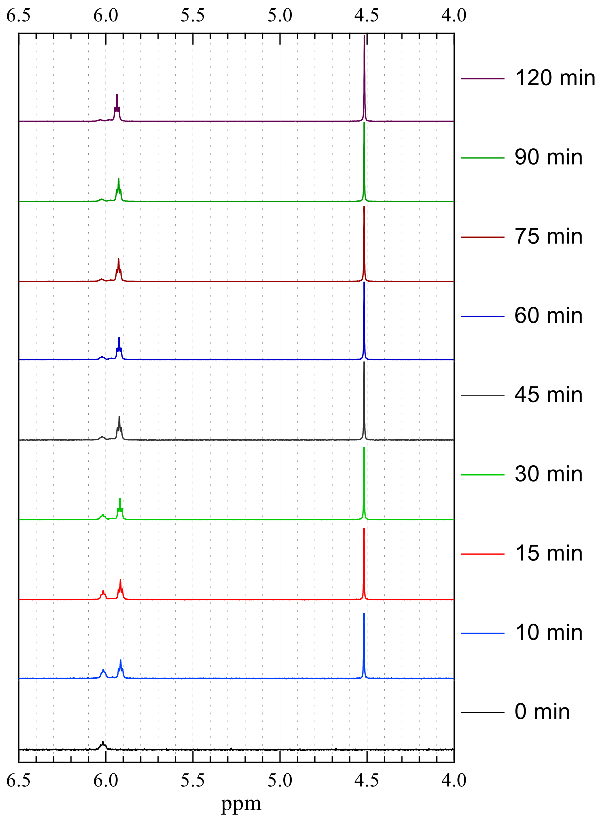

Figure S23. Expanded ^{1}H-NMR spectra of Figure S20 from 4.0 to 6.5 ppm.

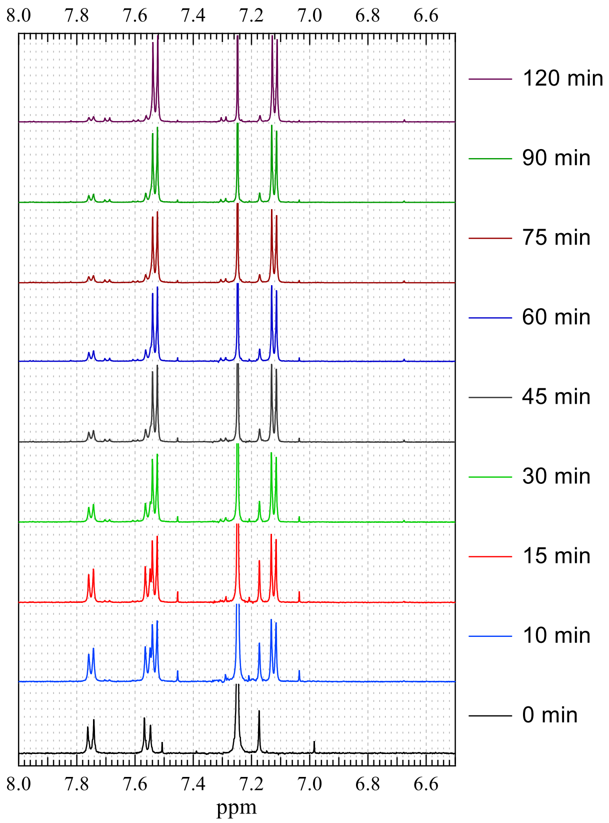

Figure S24. Expanded ^{1}H-NMR spectra of Figure S20 from 6.5 to 8.0 ppm.

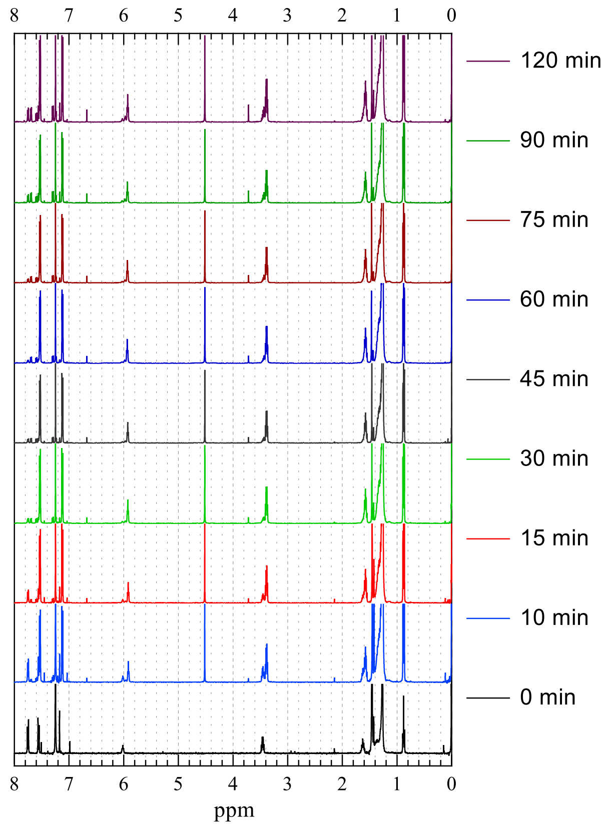

Figure S25. Photo-irradiation time-dependent ^{1}H-NMR spectra of **C14SDA** at 490 K.

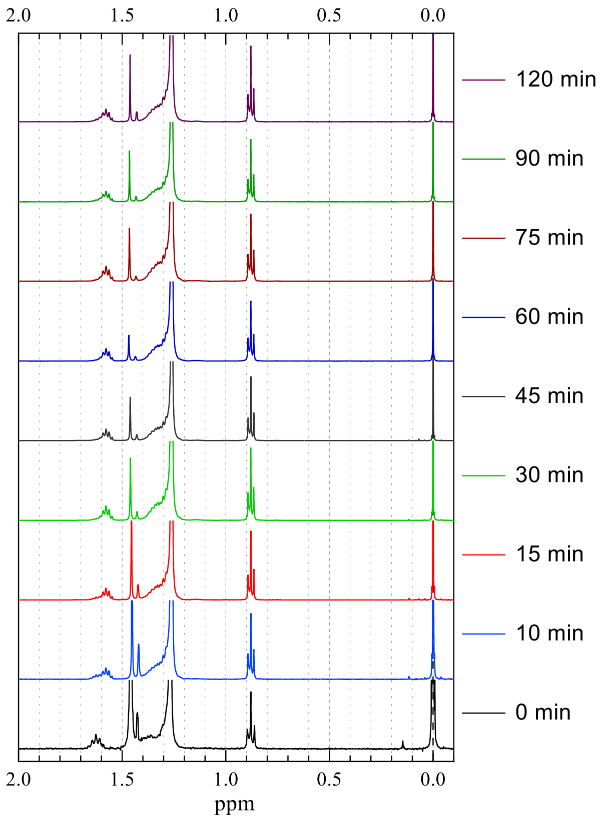

Figure S26. Expanded ^{1}H-NMR spectra of Figure S25 from 0.0 to 2.0 ppm.

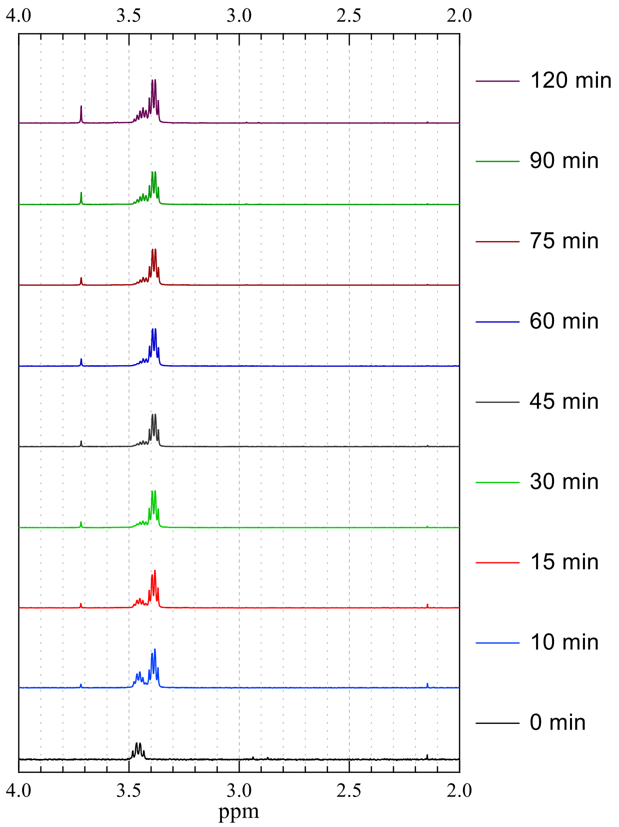

Figure S27. Expanded ^{1}H-NMR spectra of Figure S25 from 2.0 to 4.0 ppm.

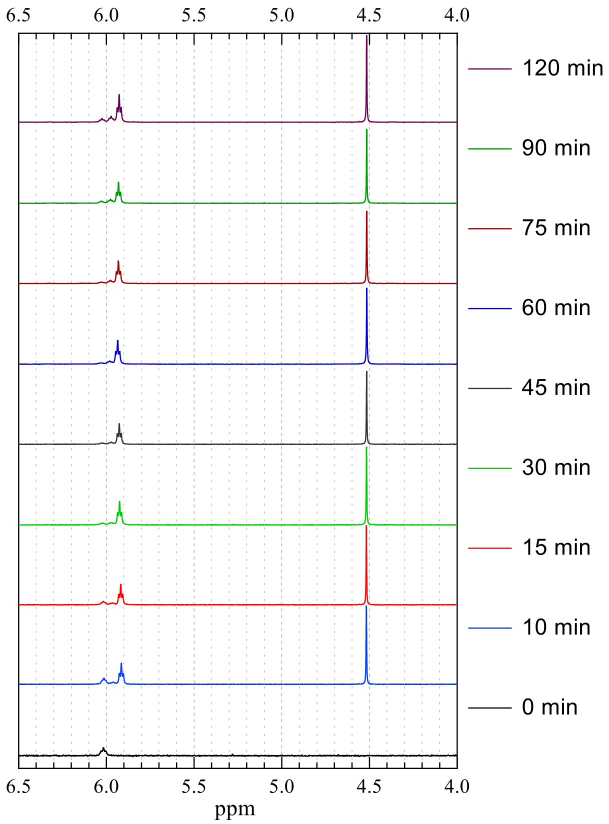

Figure S28. Expanded ^{1}H-NMR spectra of Figure S25 from 4.0 to 6.5 ppm.

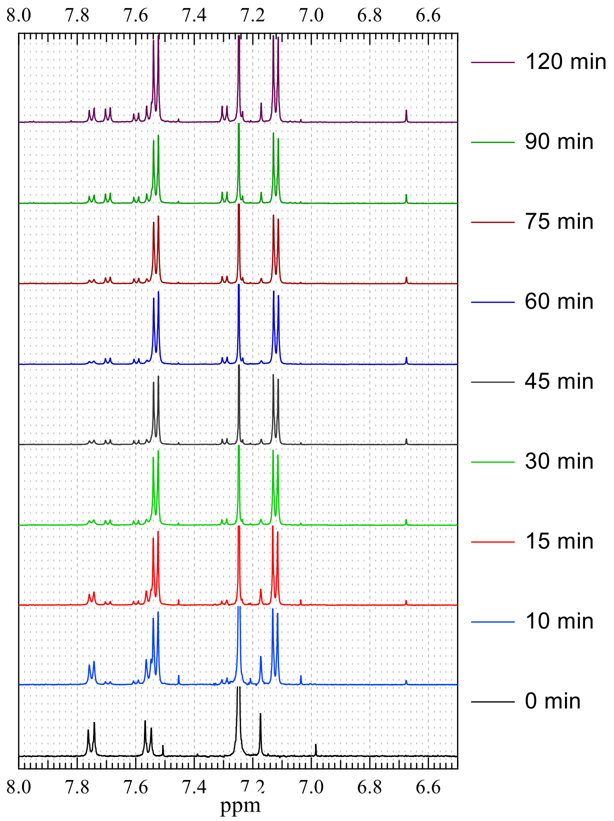

Figure S29. Expanded ^{1}H-NMR spectra of Figure S25 from 6.5 to 8.0 ppm.

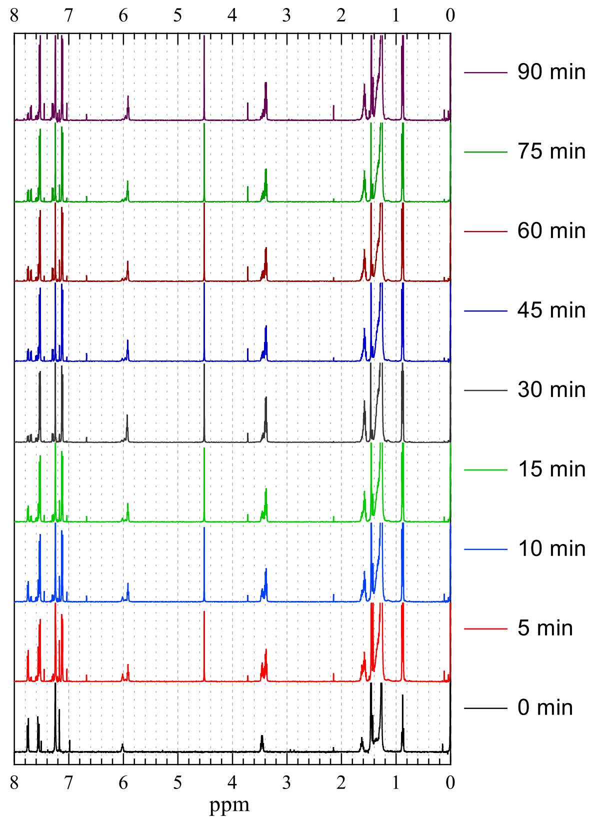

Figure S30. Photo-irradiation time-dependent ^{1}H-NMR spectra of **C14SDA** at 510 K.

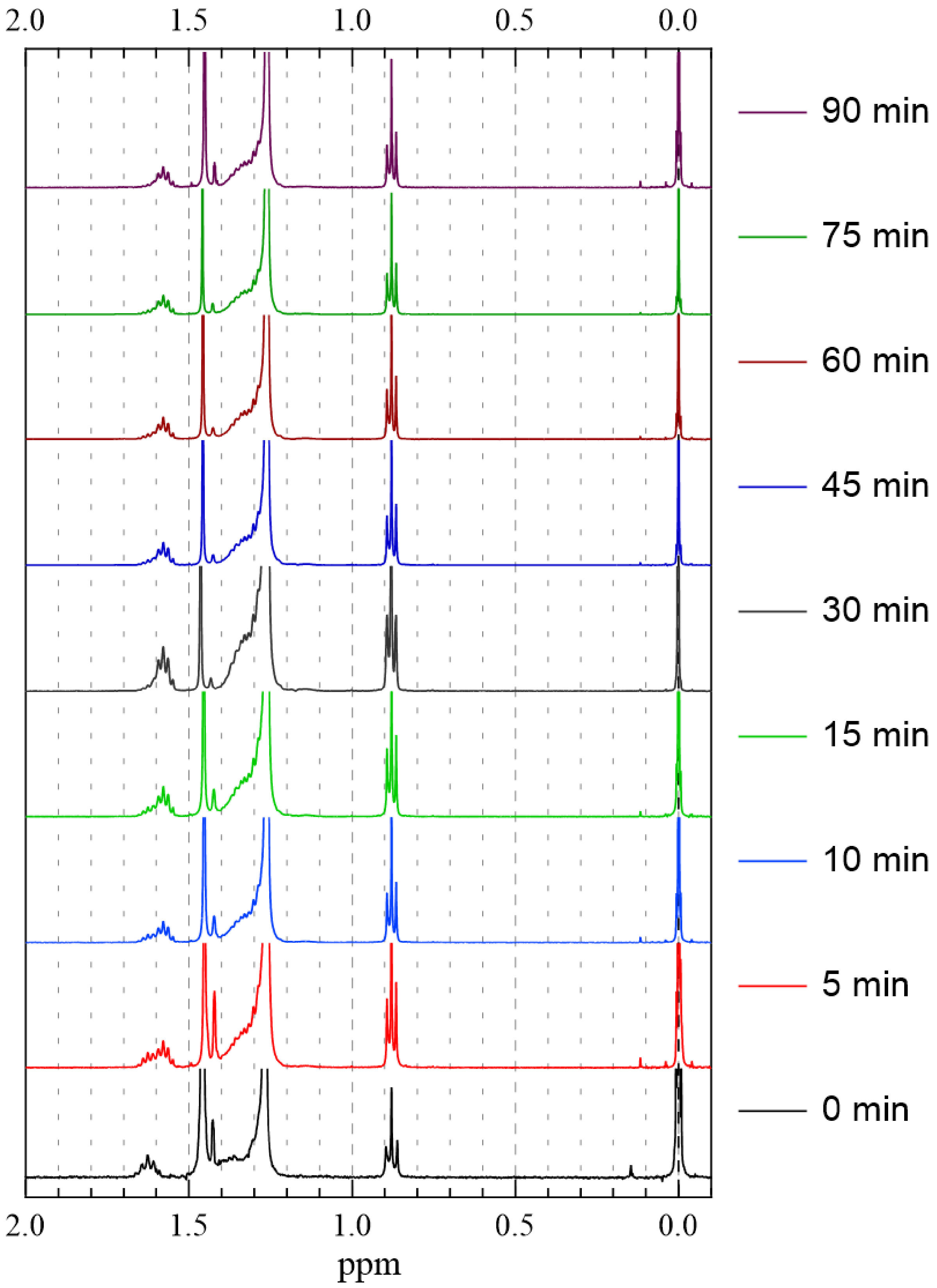

Figure S31. Expanded ^{1}H-NMR spectra of Figure S30 from 0.0 to 2.0 ppm.

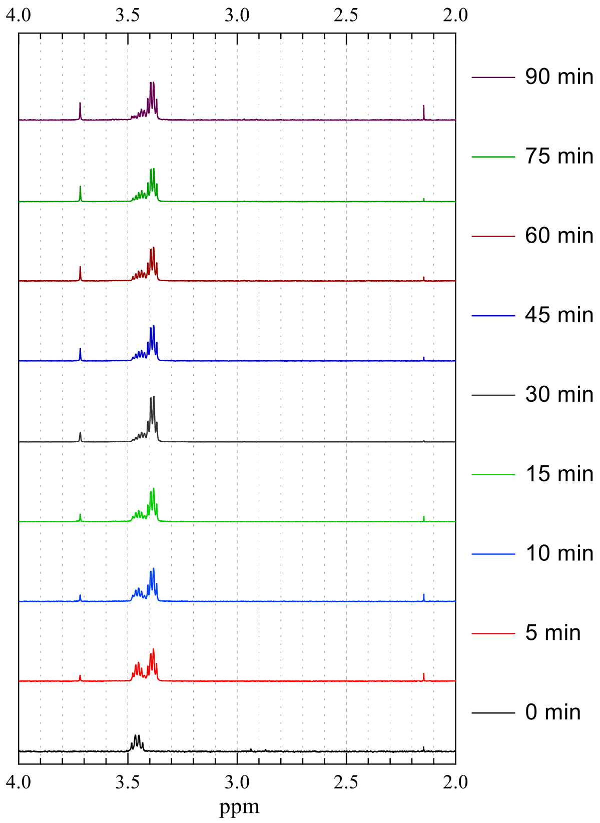

Figure S32. Expanded ^{1}H-NMR spectra of Figure S30 from 2.0 to 4.0 ppm.

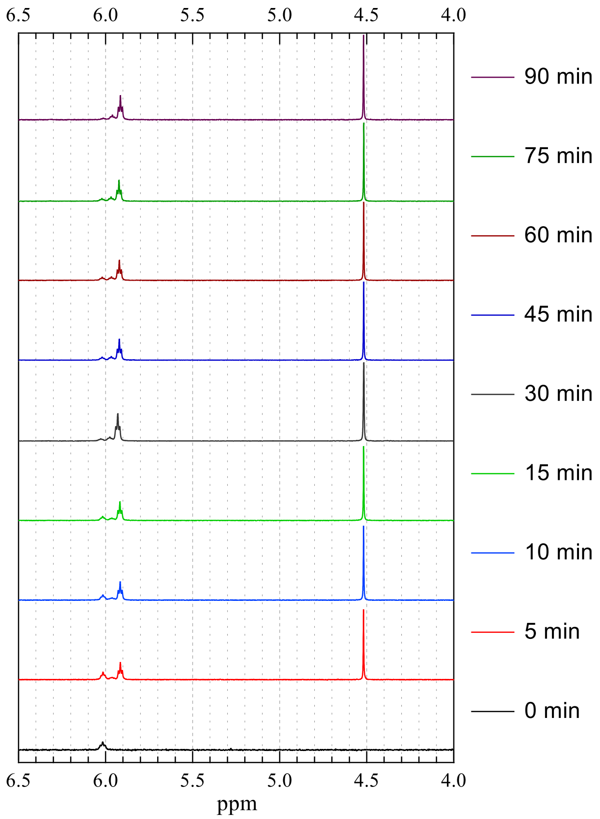

Figure S33. Expanded ^{1}H-NMR spectra of Figure S30 from 4.0 to 6.5 ppm.

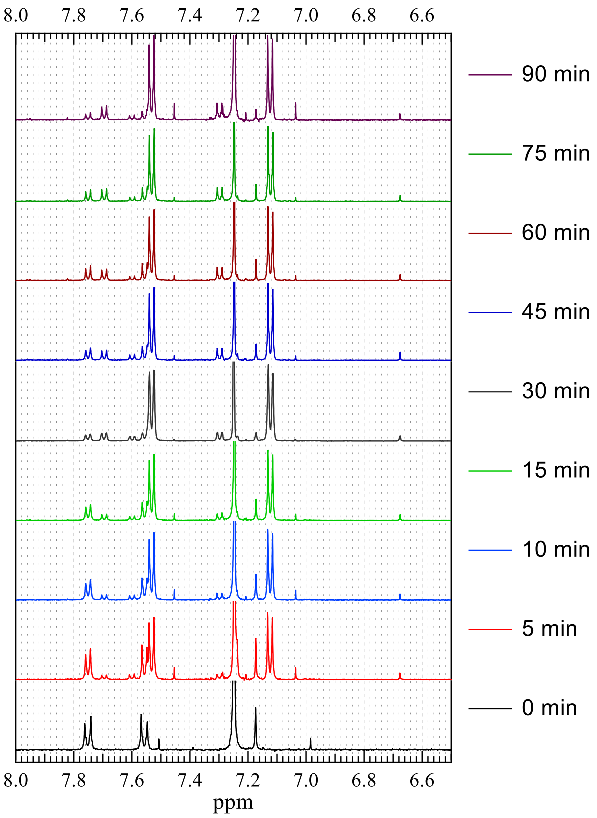

Figure S34. Expanded ^{1}H-NMR spectra of Figure S30 from 6.5 to 8.0 ppm.

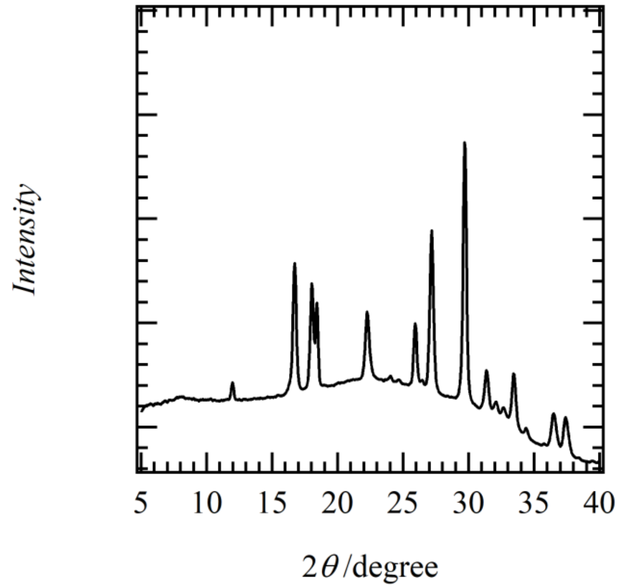

Figure S35. PXRD pattern of **C14CBDA** at 298 K.

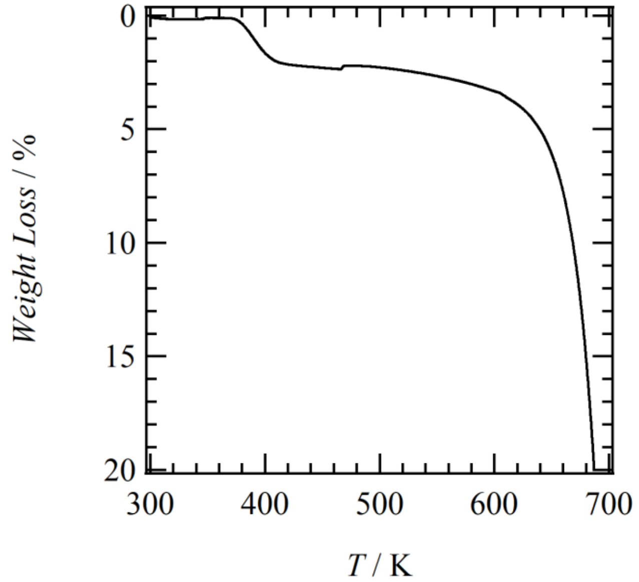

Figure S36. TG chart of **C14CBDA**•0.5($CHCl_3$).

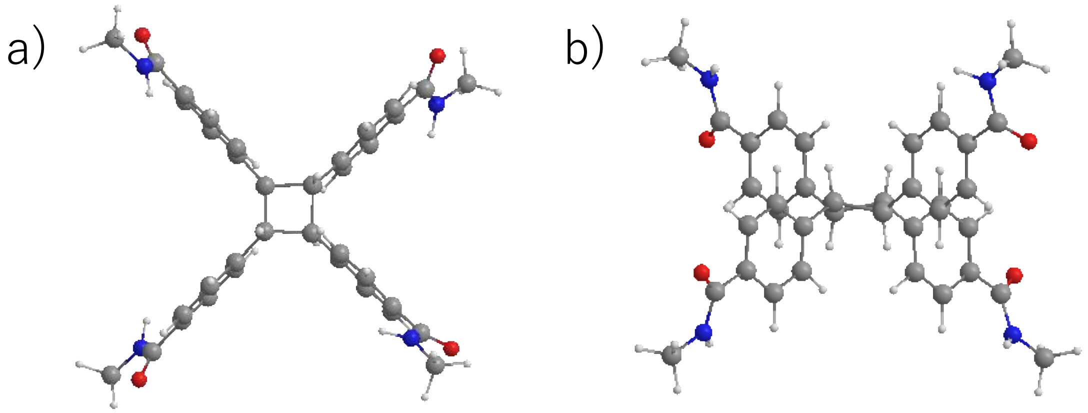

Figure S37. Optimized molecular structure of photo-dimerized **C14CBDA** using DFT calculation based on B3LYP/6-31G(d) basis set a) viewed along the cyclobutane ring and b) viewed along the direction normal to the cyclobutane-ring.

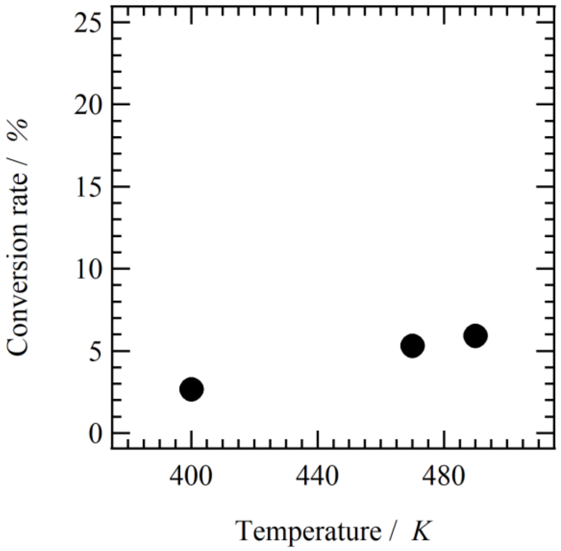

Figure S38. Temperature-dependent conversion rate of **C4SDA**.

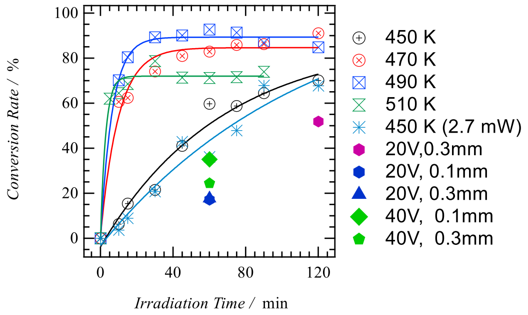

Figure S39. Temperature- and *t*-dependent conversion ratio form **C14SDA** to **C14CBDA**. additional data under the application of electric fields at 20 and 40 V using sandwich ITO cell (0.1- and 0.3-mm thickness) were included in Figure 8a. The photodimerization reaction under an electric field in the ferroelectric phase was carried out by irradiation with 4 mW cm^{-2} light at 450 K for 60 min. Under the applied electric fields (20 and 40 V) and sample thicknesses in ITO glass (0.1 and 0.3 mm) with an electrode area of 16 mm^2 using photo-irradiation power of 2.7 mW cm^{-2}.
